# Ontogeny and taxonomy of the hadrosaur (Dinosauria, Ornithopoda) remains from Basturs Poble bonebed (late early Maastrichtian, Tremp Syncline, Spain)

**DOI:** 10.1371/journal.pone.0206287

**Published:** 2018-10-31

**Authors:** Víctor Fondevilla, Fabio Marco Dalla Vecchia, Rodrigo Gaete, Àngel Galobart, Blanca Moncunill-Solé, Meike Köhler

**Affiliations:** 1 Institut Català de Paleontologia Miquel Crusafont, Sabadell, Catalonia, Spain; 2 Departament de Geologia, Universitat Autònoma de Barcelona, Bellaterra, Catalonia, Spain; 3 Museo Friulano di Storia Naturale, Udine, Italy; 4 Museu de la Conca Dellà, Isona (Lleida), Catalonia, Spain; 5 Departament de Biologia Animal i Vegetal, Universitat Autònoma de Barcelona, Bellaterra, Catalonia, Spain; 6 Institució Catalana de Recerca i Estudis Avançats ICREA, Barcelona, Catalonia, Spain; University of New England, AUSTRALIA

## Abstract

The lower Maastrichtian site of Basturs Poble (southern Pyrenees, Spain) is the first hadrosaur bonebed reported from Europe. It is an accumulation of disarticulated lambeosaurine skeletal elements, possibly belonging to *Pararhabdodon isonensis*. The sample shows high intraspecific morphological variability among many skeletal elements, suggesting the need for caution in choosing characters for phylogenetic analyses. Juvenile to adult individuals are represented in the sample, while hatchling remains are absent. Bone histology reveals that juveniles are over-represented and that the youngest individuals represented by tibia specimens were two years old. Adult individuals, with tibiae 550–600 mm long, were 14–15 years old when they died. However, individual variation in tibia length at skeletal maturity occurs within the sample, so individual maturity cannot be assumed on the basis of bone size alone. The Basturs Poble bonebed occurs within the upper part of the C31r magnetochron. Thus, lambeosaurine hadrosaurids were already present and abundant in the Ibero-Armorica Island at the end of the early Maastrichtian and *P*. *isonensis* spans the upper part of the lower Maastrichtian to the upper part of the upper Maastrichtian (upper part of C31r-lower part of C29r).

## Introduction

During the latest Cretaceous (the Campanian and Maastrichtian Ages, 83.6–66 Ma), hadrosaurid hadrosauroids were the dominant herbivorous dinosaurs of the Asian and North American landmasses [1]. Europe at that time was an archipelago of comparatively small islands that was populated by both hadrosaurids and the more primitive non-hadrosaurid hadrosauroids (the latter including *Telmatosaurus transsylvanicus* from Romania and *Tethyshadros insularis* from Italy; [2]). European hadrosauroid remains are reported from the uppermost Cretaceous of Ukraine, Bulgaria, Romania, Italy, Slovenia, Germany, Belgium and the Netherlands, but most of the hadrosauroid sites are in the French and Spanish Pyrenees [2,3]. To date, four hadrosauroid taxa (all belonging to the Lambeosaurinae) have been named from the Pyrenees: *Pararhabdodon isonensis* Casanovas-Cladellas, Santafé-Llopis and Isidro-Llorens [4]; *Arenysaurus ardevoli* Pereda-Suberbiola, Canudo, Cruzado-Caballero, Barco, López-Martínez, Oms and Ruiz-Omeñaca [5]; *Blasisaurus canudoi* Cruzado-Caballero, Pereda-Suberbiola and Ruiz-Omeñaca [6]; and *Canardia garonnensis* Prieto-Márquez, Dalla Vecchia, Gaete and Galobart [3]. However, most of the hadrosauroid material from the Pyrenees cannot be referred to species and genus level, because of fragmentary or missing-diagnostic features [2,3,7–10].

Basturs Poble (henceforth referred to as BP) is the richest of the hadrosauroid-bearing localities from the southern Pyrenees [7]. It is the largest hadrosauroid sample from a single locality and horizon in a palaeoinsular context, because the present-day Pyrenean region was part of an island (the Ibero-Armorican Island) within the European Archipelago during latest Cretaceous times [11].

In the absence of evidence supporting the presence of distinct species, the BP hadrosauroid sample initially was referred to a single hadrosauroid species at different ontogenetic stages [12,13]. Prieto-Márquez et al. [14] provisionally referred this species to the Lambeosaurinae on the basis of features of the jugal and maxilla, and suggested that it could be *Koutalisaurus kohlerorum*. However, this taxon later was demonstrated to be invalid, because its holotype and unique specimen (an incomplete dentary) lacks autapomorphies or an autapomorphic combination of characters [3].

Blanco et al. [15] identified two differently-sized dentary morphotypes in the BP sample by multivariate analysis. A monotaxic sample was ruled out on the basis of the different allometric trajectories of the small and larger dentary morphotypes, and the small "morphotype 3" was referred to a distinct, basal and possibly dwarf hadrosauroid taxon.

This paper deals with the osteology, histology and taxonomy of the hadrosauroid specimens from the BP bonebed. Our aim is to establish: 1) whether the presence of more than one taxon can be detected through morphological and histological study; 2) the size distribution and the frequency of ontogenetic stages within the assemblage; 3) the systematic affinity of that taxon/ those taxa at the clade level (non-hadrosaurid hadrosauroid, hadrosaurid, saurolophine or lambeosaurine) or at the species level.

The relative abundance of fossil remains allowed us to undertake a histological study of the BP hadrosauroids in order to identify the ontogenetic stage (juvenile, subadult, or adult) of differently-sized individuals and hopefully to establish the presence of one or more species in the sample. In order to achieve this, we constructed a growth curve using the most-represented skeletal elements.

Institutional abbreviations—**IPS**, Institut de Paleontologia Miquel Crusafont, currently **ICP**, Institut Català de Paleontologia ‘Miquel Crusafont’, Sabadell, Spain; **MCD**, Museu de la Conca Dellà, Isona, Spain; **MPZ**, Museo Paleontológico de la Universidad de Zaragoza, Zaragoza, Spain; **TMP**, Royal Tyrrell Museum of Paleontology, Drumheller, Canada; **ROM**, Royal Ontario Museum, Toronto, Canada.

## Material and methods

Field work at the BP locality was undertaken in 2001–2007 and 2009–2011 by the MCD and the IPS-ICP. All necessary excavation permits were obtained for the described study, which complied with all relevant regulations. They were granted by the Catalan Government (Departament de Cultura, Generalitat de Catalunya). Permit numbers are: 494 K0121 N355, 437 K121 N355, 437 K121 N355 3793, 470 K121 N355/-2011-1-6910. The fossil specimens and thin sections involved in this study are deposited and accessible to other researchers at the MCD (Isona, Spain) and the ICP (Bellaterra, Cerdanyola del Vallès, Spain), respectively. Permission was granted to the authors for accessing the collections at the MCD, ICP and the TMP (Drumheller, Canada) in order to examine the materials housed at these institutions.

The portion of the prepared BP sample that can be referred to hadrosauroids with confidence is composed of 270 skeletal elements, which mostly are incomplete or fragmentary. Thirty-six specimens are from the skull and lower jaw (17 are cranial elements and 19 are dentaries); three are isolated teeth; 131 or 132 are axial elements (21 belonging to cervical vertebrae, five to dorsal vertebrae, 11 to the sacrum [including three partial sacra], 48 to caudal vertebrae, 15 to unidentified vertebrae, 14 to dorsal ribs, seven or eight to chevrons, and ten to segments of ossified tendons); eight are girdle elements (six from the shoulder and two from the pelvic girdle); and 92 are limb bones (10 humeri, five ulnae, three radii, five metacarpals, one manual phalanx, 23 femora, 20 tibiae, six fibulae, 10 metatarsals, and nine pedal phalanges).

From among these specimens, we present 102 skeletal elements that provide taxonomic or ontogenetic information: one maxilla (MCD-5099); one jugal (MCD-5100); one frontal (MCD-4869a); one quadrate (MCD-5278); one exoccipital (MCD-4851a); 19 dentaries (MCD-4779, MCD-4944, MCD-5007, MCD-5097, MCD-4942a, MCD-4942b, MCD-5098, MCD-5108, MCD-4726a, MCD-5096, MCD-4836, MCD-5012, MCD-4945, MCD-5008, MCD-4963, MCD-4743, MCD-4833, MCD-4946 and MCD-4744); one neural arch of a dorsal vertebra (MCD-4891); two dorsal ribs (MCD-4848 and MCD-4865); four isolated sacral centra (MCD-4966, MCD-4995, MCD-4897 and MCD-4851b); one synsacrum (MCD-4745); one neural arch of a proximal caudal vertebra (MCD-4938); one chevron (MCD-4850); four scapulae (MCD-4738, MCD-4826, MCD-4781 and MCD-4717); one ischium (MCD-4881); 10 humeri (MCD-4825, MCD-4706, MCD-4845, MCD-5021, MCD-4746, MCD-4818, MCD-5376a, MCD-5009, MCD-4846 and MCD-5110); three ulnae (MCD-4724, MCD-4725 and MCD-4841); one radius (MCD-4752); 23 femora (MCD-4742, MCD-4941, MCD-5107, MCD-5011, MCD-4754, MCD-4723, MCD-4800, MCD-4804, MCD-4801, MCD-4708, MCD-4729, MCD-4802, MCD-4704, MCD-5104, MCD-4702, MCD-4987, MCD-4722, MCD-4998, MCD-4983, MCD-4783, MCD-4892, MCD-5369 and MCD-5370); 20 tibiae (MCD-5109, MCD-4728, MCD-4958, MCD-4918, MCD-4701, MCD-4920, MCD-4719, MCD-5105, MCD-4705, MCD-4784, MCD-4771, MCD-4886, MCD-4796, MCD-4986, MCD-4799, MCD-4882, MCD-4956, MCD-4721, MCD-7144 and MCD-5106); and six fibulae (MCD-4889, MCD-4912, MCD-4715, MCD-4795, MCD-4741 and MCD-4985).

The lengths of incomplete or fragmentary dentaries (Table A in S1 Text) were estimated by comparing and scaling with the most complete specimens (MCD-5007, MCD-5008, MCD-4945 and MCD-4744) and with the right dentary of *Corythosaurus intermedius* in Parks ([16]: pl. 3, fig 2). The measurements (lengths and angles) taken on dentaries are approximate, because they are biased by the following factors: 1) the margins and extremities of the dentaries and their processes (e.g., the coronoid process) are often damaged or missing; 2) the bones are affected by crushing (caused by lithostatic pressure and/or by trampling; [12]) and sometimes by slight deformation; 3) all the dentaries were broken into fragments when collected and were reassembled with degrees of inaccuracy (with margins between the various glued fragments that evidently do not match) and subjected to interpretative restoration in the laboratory. The length of the humerus was measured from the top of the caput humeri to the end of the distal condyles; the deltopectoral crest length was measured as in Prieto-Márquez ([17]: character 219, http://www.morphbank.net/Show/?id=461721). The total lengths of incomplete humeri were estimated by comparing and scaling with the most complete ones (MCD-4706, MCD-4818 and MCD-5009). The estimated total lengths of incomplete femora (Table B in S1 Text) and tibiae (Table C in S1 Text) were calculated by comparing and scaling with the almost complete specimens, MCD-4728 and MCD-5011, respectively. For the very fragmentary largest tibia (MCD-5109), the circumference at mid-shaft was calculated using a bone circumference vs. bone length linear regression (see below). For measurements of angles and other parameters taken on the bones and teeth, the references are the methodological explanations in Prieto-Márquez [17] as reported in MorphBank, if not otherwise specified.

The most-represented elements (dentaries, femora and tibiae) were used for size frequency analysis. Tibiae and femora were used for histological analysis.

Tibiae were sectioned at mid-shaft (Fig A in S2 Text). When the mid-shaft was missing or crushed, we sectioned the preserved portion nearest the mid-shaft. This was the case for tibia MCD-4719, from which two thin sections were prepared. The diaphyses of the femora were sectioned just distal to the fourth trochanter in MCD-4802, MCD-5104 and MCD-4723. The sampling locations for the other specimens are shown in Fig A in S2 Text. In order to recompose the cut bone, casts were made of the regions to be sectioned.

After sectioning, the bones were reconstructed and the casts were painted. To preserve the specimens, each bone was embedded in epoxy resin (Araldite 2020) before sectioning. Thin sections were made using standard histological procedure (see S2 Text) and were observed under transmitted and polarized light (Leica DM 2500P, Zeiss AxioScope.A1), using a λ filter when necessary.

We identified the perimeters of the periosteum and medullary cavity, the growth cycles/lines of arrested growth (LAG) and the remodeled areas. We made sketches using Adobe Illustrator CS5. Clear transitions in LAGs, annuli, growth cycles in alternating slow- and fast-growth zones, and major decreases in vascularity, were considered to be annual growth marks even when they were discontinuous. The reliability of growth cycles as annual equivalents of LAGs was tested by Hübner [18]. In addition, growth cycles often are preceded by LAGs in the BP specimens. LAGs in the BP specimens often are discontinuous because of regional changes in growth rate and local tissue destruction by microorganisms. Additionally, the innermost growth cycles in older BP individuals are obliterated to a lesser or greater extent by formation of secondary osteons and were partially reabsorbed by medullary expansion. LAGs within the External Fundamental System (EFS) of adult bones were difficult to trace, allowing only rough estimation of the time span covered [19]. We avoided using radii for calculating annual growth or LAG circumferences [20,21] because of the locally incomplete rest marks. Instead, employing image compositions of the whole cross-section by Adobe Photoshop CS5 software, we scaled the outer bone perimeter down to fit the identified growth cycle elements, and thus, to reconstruct the ontogeny of growth cycles within the cortex (Fig B in S2 Text).

Bone circumference at hatching was taken from Cooper et al. [21]. Annual growth was calculated by measuring the reconstructed annual rest marks and the periosteal margin using ImageJ software (Figs B–C and Table A in S2 Text). To increase the accuracy of our measurements, the bone section surfaces were reconstructed using Photoshop CS5 in two specimens (MCD-4920 and MCD-4728) in which fragmented areas could be aligned. The differences in surface values of original and reconstructed MCD-4728 were minimal and the reconstructed areas provided growth cycles that were very similar in size and shape to those of the smaller juvenile specimens. However, superimposition of early juvenile growth cycles on the reconstructed specimen MCD-4920 was not possible because of deformation of the bone, even after mending the fragments using Photoshop CS5. Nevertheless, it was possible to measure the annual increase in circumference of MCD-4920, and it fitted that of MCD-4918 over the interval between three and eight years, suggesting an age of three years for this specimen at the first preserved growth cycle. For tibia MCD-5109, the nature of the sample (a core drilled in the distal end of the shaft; Fig A10 in S2 Text), the extensive remodeling of the bone matrix, and the missing outermost portion of the EFS, preclude reconstruction of growth trajectory, age at asymptotic size, or age at death. Therefore, a minimum age was calculated based on the growth cycle count. When possible, reconstruction of the less preserved innermost rest marks in older specimens was evaluated by superimposing these innermost rest marks on the early ontogenetic stages of juveniles. Von Bertalanffy’s [22] model, which considers body size as a function of age, was used to calculate the mean growth curves with PAST software. Von Bertalanffy’s model as applied to the BP values is: Circumference = a*[1-(b*exp(- c*time))], where “a” is the asymptotic length at which growth is zero, “b” is a constant, and “c” is the growth rate. Circumference = 175.48*[1-(0.85013*exp(0.42901*Time))]. The logarithmic model for the dental battery length vs. number of alveoli plot, calculated with SPSS, is: Number of alveoli = -16.501 + (16.887 * ln battery length). The reverse model is: Number of alveoli = 47.650 - (263.484/battery length).

Clade names, phylogenetic definitions, and clade synapomorphies follow the systematic revision of hadrosaurid relationships by Prieto-Márquez [17] and that of lambeosaurines by Prieto-Márquez et al. [3]. Accordingly, the Hadrosauroidea are defined as *Hadrosaurus foulkii* and all taxa more closely related to it than to *Iguanodon bernissartensis*. The Hadrosauridae consist of the last common ancestor of *H*. *foulkii*, *Edmontosaurus regalis*, *Saurolophus osborni* and *Lambeosaurus lambei* and all its descendants. Likewise, the Saurolophidae consist of the last common ancestor of *S*. *osborni* and *L*. *lambei*, and all its descendants. The Saurolophinae consist of *S*. *osborni* and all taxa more closely related to it than to *L*. *lambei* or *H*. *foulkii*. The Lambeosaurinae consist of *L*. *lambei* and all taxa more closely related to it than to *H*. *foulkii*, *S*. *osborni*, or *E*. *regalis*.

**Fig 1:**
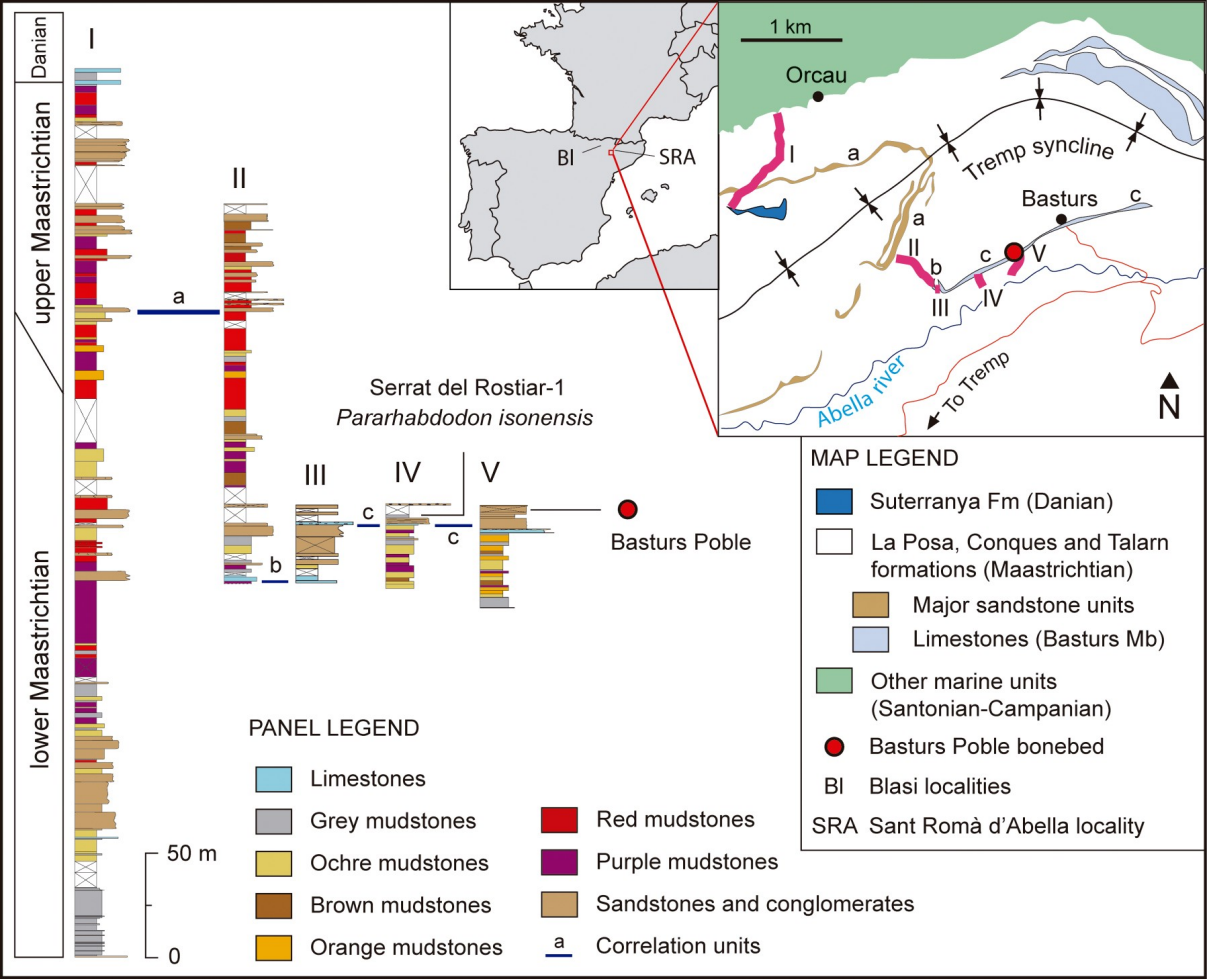
Geographical, geological and stratigraphic context of the Basturs Poble bonebed. Stratigraphic correlation is modified from Blanco et al. [24], Riera et al. [25], Vila et al. [26] and Fondevilla et al. [32]. The geographical map is modified from Vila et al. [26], and the geological map is redrawn from Riera et al. [25]. Chronostratigraphic data are after Díez-Canseco et al. [31] and Fondevilla et al. [32]. The coeval site of Serrat del Rostiar-1 is also represented in the correlation panel. The locations of Blasi-1,-3 (type localities of *Blasisaurus canudoi* and *Arenysaurus ardevoli*, respectively) and Sant Romà d’Abella (type locality of *Pararhabdodon isonensis*) are shown in the geographical map.

The stratigraphic section I (Fig 1) is modified from Oms et al. [23] and Blanco et al. [24]; sections II and V are modified from Riera et al. [25]; sections III and IV are modified from Vila et al. [26] and Riera [27], respectively.

## The Basturs Poble site

The Basturs Poble locality is situated in the easternmost part of the Tremp Syncline (known as Conca Dellà) in the Catalonian Pre-Pyrenees (Fig 1). It was discovered in the late 1990s by Marc Boada and is located 300 m west of the village of Basturs (Isona i Conca Dellà Municipality, Lleida Province, Catalonia, Spain). The name (poble = village) distinguishes it from the nearby dinosaur egg-locality known in the literature by the name of the village [28]. The BP U.T.M. coordinates are X: 335283, Y: 4668339, Z: 620 m.

The fossil-bearing section belongs to the latest Cretaceous, fluvial-deltaic Conques Formation of the Tremp Group, also known in the literature as the Lower Red Garumnian [29,30]. The fossil-bearing section immediately overlies the lacustrine Basturs Limestone Member [25] and, according to the age constraints provided by Díez-Canseco et al. [31] and Fondevilla et al. [32], occurs in the upper part of the C31r magnetochron and is late early Maastrichtian in age (Fig 1).

The BP section preserves a 1.5-m-thick composite bonebed consisting of two overlying fossil-bearing layers [12,27]. The lower layer is a palaeosol consisting of fine-grained sandstone with edaphic carbonate nodules and root bioturbation (mottling). The upper layer is also composed of fine-grained sandstone, but it was produced by a low-energy mass transport that reworked the upper part of the palaeosoil and the bones contained at the top of the latter [12,27]. Skeletal remains from both horizons display similar weathering and abrasion features [12], are encrusted by iron oxide, and often are incomplete or fragmentary. The lower accumulation is considered to be an in situ, attritional concentration without significant bone transport, but with total skeletal disarticulation, element fragmentation and a morphological bias in the preservation of skeletal elements [12].

The total BP sample consists of about one thousand disarticulated skeletal remains. Dinosaur bones and teeth account for most of the sample (about 95%). Nearly all identified dinosaur skeletal elements belong to hadrosauroids. Skeletal elements of other dinosaur clades have not been identified positively, apart from three theropod teeth collected during the 2010 field work [7]. Other vertebrates identified in the sample are small-sized crocodyliforms that are represented by teeth, jaws and vertebrae. A few eggshell fragments referred to the theropod ootaxon *Prismatoolithus trempii* [33], remains of the terrestrial gastropod *Lychnus*, and abundant coal fragments have also been collected.

## Osteological data

Most of the examined hadrosauroid skeletal elements have no features that could be used to refer them to a less inclusive taxon. Here we report only information that has taxonomic and ontogenetic relevance.

### Taxonomic information and comparison

In general, the BP remains under study cannot be immediately and clearly referred to two or more distinct taxa on a morphological basis, as already noted by Martín et al. [12] and Gaete [13]. This is also the case with the hadrosaurid bonebeds in North America [19] and Asia [34] that are considered to be monotaxic in the absence of evidence to the contrary. However, Blanco et al. [15] identified two different taxa (a small and possibly dwarf basal hadrosauroid and a larger lambeosaurine) in the BP sample on the basis of the morphometrics of the dentaries. The taxonomic data from the whole BP sample must be evaluated in order to support or refute Blanco et al.’s [15] hypothesis.

Cranial elements—The taxonomically most informative elements in the hadrosauroid skeleton are those from the skull [3,17,35], which unfortunately are rare in the BP sample.

**Fig 2:**
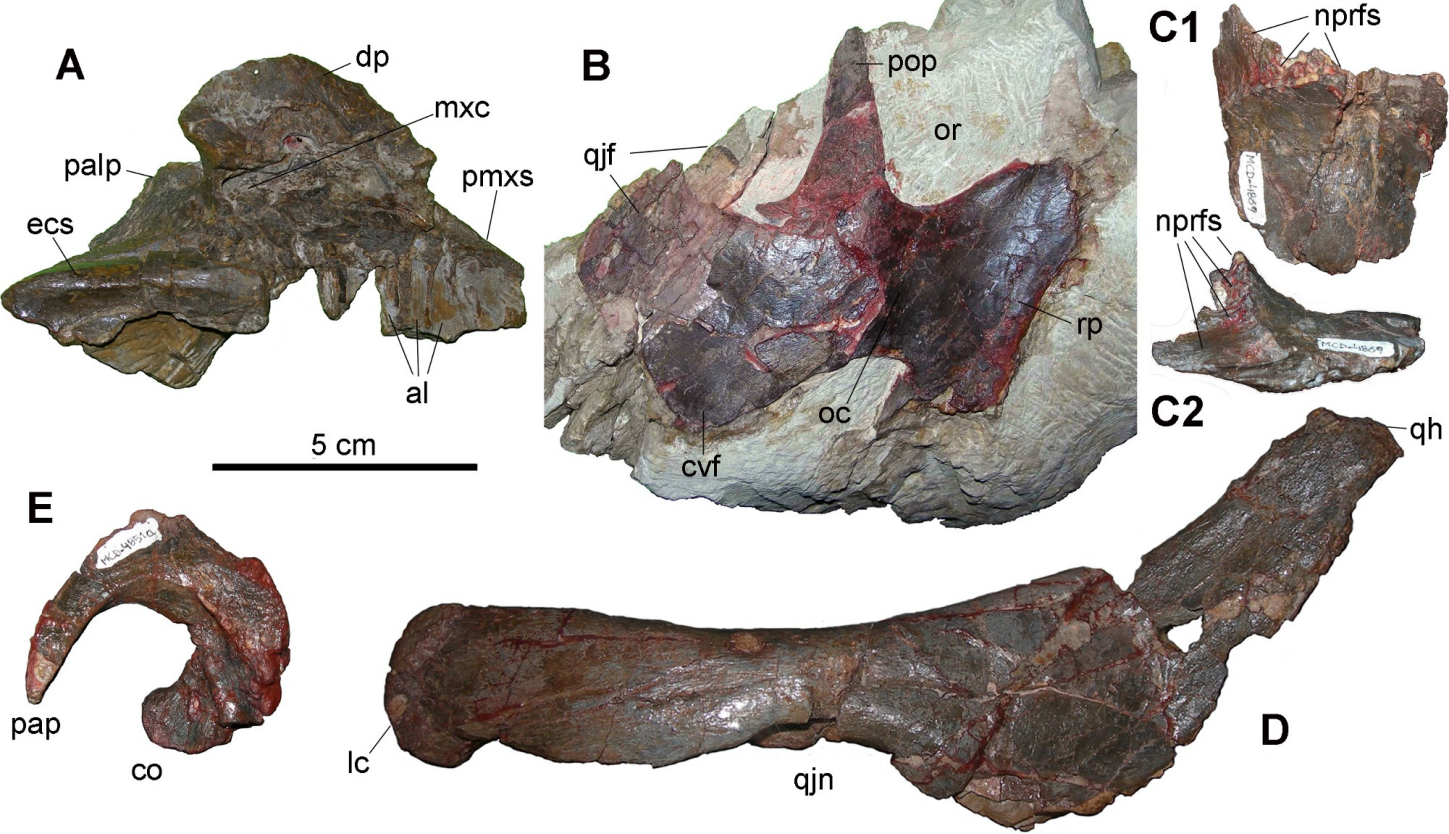
Skull elements from Basturs Poble bonebed. (A) Right maxilla (MCD-5099), lateral view. (B) Right jugal (MCD-5100), lateral view. (C) Left frontal (MCD-4869a) in dorsal (C1) and lateral (C2) views. (D) Right quadrate (MCD-5278), lateral view (rotated 90° clockwise with respect to its anatomical orientation). E) Right exoccipital (MCD-4851a), anterior view. Abbreviations: al, alveolus; cef, cerebral fossa; co, condyloid; cvf, caudoventral flange of jugal; dp, dorsal process of maxilla; ecs, ectopterygoid shelf; lc, lateral distal condyle of quadrate; mxc, maxillary canal; nprfs, articulation surface for prefrontal and nasal; oc, orbital constriction; or, orbit; palp, palatine process; pap, paroccipital process; pmxs, premaxillary shelf; popo, postorbital process; qh, quadrate head; qjf, quadratojugal flange; qjn, quadratojugal notch; rp, rostral process. Scale bar equals 5 cm.

As noted by Prieto-Márquez et al. [14], the small right maxilla MCD-5099 (Fig 2A) is proportionately short and its dorsal process is tall, as in lambeosaurines [17,35]. The dorsal profile of the process is rounded with the apex in the central part of the element, but its dorsal margin is damaged. No rough or depressed jugal facet can be distinguished on the damaged lateral surface of the dorsal process, so the diagnostic features of the Tsintaosaurini [3] cannot be verified. The toothless alveoli are comparatively large considering the size of the bone (their mesiodistal width measured between the mid-points of the alveolar septa is 5–5.5 mm), but this could be related to immaturity.

The right jugal MCD-5100 (Fig 2B) is more informative. It is rostrocaudally short and robust. The ratio between the maximum rostrocaudal length and the minimum jugal neck height is 3.4. Its rostral process is rostrally truncated and dorsoventrally expanded. The rostral margin of the process is straight. Its shape suggests a narrow, tall and vertical articular facet for it on the maxilla, which consequently had to have a tall dorsal process to accommodate it. The orbital and infratemporal constrictions, and the caudoventral flange, are as deep as in some lambeosaurines (e.g., *Amurosaurus riabinini*; [36]). The combination of all these characters indicates that the jugal belongs to a lambeosaurine hadrosaurid [17,35]. Absence of the rostral apex, combined with a straight rostral margin of the rostral process of the jugal, is a feature that is restricted to the lambeosaurine *Hypacrosaurus altispinus*, *Olorotitan arharensis* and possibly *Tsintaosaurus spinorhinus* (see [3]: SI). The rostral process is anterodorsally inclined at about 60° with respect to the body of the jugal. A similar sloping possibly occurs in the basal lambeosaurine *Aralosaurus tuberiferus* (see [37]: fig 4A).

The rostral process of the left jugal MPZ 99/667, holotype of *Blasisaurus canudoi*, has a slight rostral inclination and possibly a straight rostral margin too, but its rostral margin is damaged (see [3,6]). The partially preserved quadratojugal flange of MCD-5100 appears to be hook-shaped like that of the jugal of *B*. *canudoi*. However, the quadratojugal flange of *B*. *canudoi* is taller and more slender than that of MCD-5100. The caudoventral flange is more slender as well, and is asymmetrical and caudally skewed. The broad notch between the caudoventral flange and the quadratojugal process occurring in the jugal of *B*. *canudoi* and many saurolophines (e.g., *Gryposaurus notabilis*, *Edmontosaurus* spp., *Brachylophosaurus canadensis* and *Maiasaura peeblesorum*; [35]) probably is not present in MCD-5100. This notch is also lacking in the jugals of the lambeosaurine *Charonosaurus jiayinensis* and *Olorotitan arharensis*. The partially preserved left jugal MPZ2011/1 referred to *Arenysaurus ardevoli* by Cruzado-Caballero et al. [38] also differs from MCD-5100, because its rostral process is not inclined anterodorsally (see [38]: fig 2).

**Fig 3:**
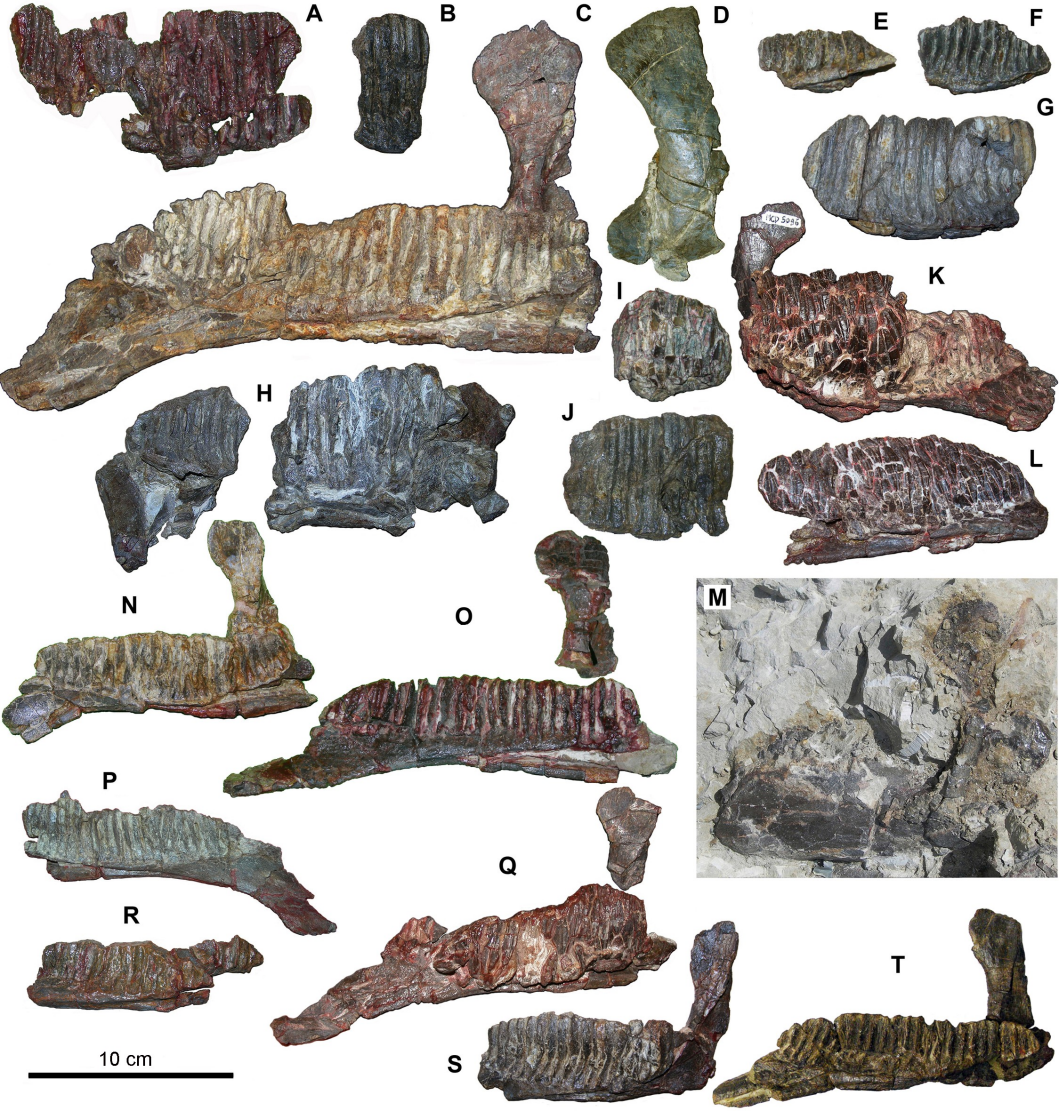
Dentaries from Basturs Poble bonebed (in lingual view, if not specified otherwise). (A) MCD-4779. (B) MCD-4944. (C) MCD-5007. (D) MCD-5097. (E) MCD-4942a. (F) MCD-4942b. (G) MCD-5098. (H) MCD-5108. (I) MCD-4726a. (K) MCD-5096. (J) MCD-4836. (L) MCD-5012. (M) MCD-5012 in the field with the impression of the coronoid process (labial side). (N) MCD-4945. (O) MCD-5008. (P) MCD-4963. (Q) MCD-4743. (R) MCD-4833. (S) MCD-4946. (T) MCD-4744. Scale bar equals 10 cm.

The left frontal MCD-4869a (Fig 2C) is short rostrocaudally and its rostral margin bears a dorsally facing articular surface that is bounded caudally by a raised rim. A dorsal orientation of the articular surface for the nasal occurs in the frontals of basal lambeosaurines and Lambeosaurini and is related to the presence of a cranial crest ([39]: fig 9). However, the morphology of the articular surface of MCD-4869a does not match exactly those of the known lambeosaurines [39]. The frontals of *Arenysaurus ardevoli* have an articular surface for the nasal (the frontal platform) that is much longer rostrally ([5]: fig 3A). Caudal to the raised rim of the rostral articular surface, the frontal MCD-4869a is slightly domed dorsally, as is usual in young lambeosaurines [17,35,40].

The caudal margin of the dorsal third of the right quadrate MCD-5278 (Fig 2D) is strongly curved caudally, relative to the ventral half of the quadrate, forming an angle of 130°. A curvature of less than 150° is characteristic of lambeosaurines, although it was convergently-acquired in the non-hadrosauroid iguanodontian *Iguanodon bernissartensis* and the saurolophine *Shantungosaurus giganteus* (see [17]). The quadratojugal notch is displaced ventrally and the centre of the notch lies ventral to the mid-height of the quadrate, as in many basal hadrosauroids [41,42] and saurolophine hadrosaurids [17], but also as in the lambeosaurine *Sahaliyania elunchunorum* (see [43]).

Dentaries—The BP sample includes 19 dentaries (Fig 3). In dorsal view, the dentaries are sigmoid (e.g., MCD-4743 and MCD-4963), although this is less evident in some specimens (MCD-4945, 4946, 5012 and 5096), probably because of crushing and incomplete preservation of the rostral and caudal extremities. The larger MCD-5007 is slightly sigmoid, so this feature is independent of size. Unlike the other dentaries, MCD-5008 does not seem to be sigmoid. The occlusal plane (sensu [17]: character 48, http://www.morphbank.net/Show/?id=461250) follows the curvature of the dentary; it is parallel to the lateral side of the dentary, in MCD-4945, MCD-4946, MCD-4963, MCD-5007, MCD-5008 and MCD-5096. However, this character state is obviously biased by crushing. The exact extent of the proximal edentulous slope of the dentary cannot be known because the rostrodorsal margin of the dentary is damaged in all specimens. Whatever the case, the proximal edentulous slope was undoubtedly quite short, with a ratio between its length and the distance between the rostral-most tooth position and the caudal margin of the coronoid process ([17]: character 33, http://www.morphbank.net/Show/?id=461235) that is less than 0.20 in MCD-4945, MCD-4963 and MCD-5007, at least. A ratio less than 0.20 occurs in non-hadrosaurid hadrosauroids, but also in the lambeosaurine *Arenysaurus ardevoli* and *Blasisaurus canudoi* (see [3]: SI). The angle of deflection of the rostral ventral margin of the dentary (measured as in [17]: character 26, http://www.morphbank.net/Show/?id=461238) is quite variable, ranging from 13º (MCD-4744) to 30º (MCD-4945). It is 28º in MCD-5007; 25º in MCD-4963; 23º in MCD-5096; and 16º in MCD-5008. This range corresponds to a variety of hadrosauroids, including basal forms (the angle is 16º, 24º and 26º in *Mantellisaurus atherfieldensis*, *Bactrosaurus johnsoni* and *Telmatosaurus transsylvanicus*, respectively), saurolophines (the angle is 17.5º-26º, 22º-23º and 23º-29º in *Brachylophosaurus canadensis*, *Maiasaura peeblesorum* and *Gryposaurus notabilis*, respectively) and lambeosaurines (the angle being 15°, 21° and 22°-32° in the lambeosaurine *Parasaurolophus walkeri*, *Charonosaurus jiayinensis* and *Corythosaurus casuarius*, respectively; [17], http://www.morphbank.net/Show/?id=461238). However, the angle measurements in the BP dentaries are biased to various degrees by the factors listed above, so they must be considered with caution. The deflection always begins slightly rostrally to the middle of the dental battery (MCD-4743, MCD-4744, MCD-4945, MCD-4963, MCD-5007, MCD-5008 and MCD-5012); MCD-5096 might be an exception as the deflection seems to begin at mid-point). The deflection begins near the middle of the dental battery in some non-hadrosaurid hadrosauroids, but also in many lambeosaurines (including *A*. *ardevoli* and *B*. *canudoi*; [3]: SI). In other lambeosaurines, it occurs in a slightly more rostral position ([3]: character 27, SI). However, variability among the character states within a single species when a broad sample is available (e.g., in the saurolophine *B*. *canadensis*; [3]: SI) suggests that this character is intraspecifically variable.

The bulging of the ventral margin of the dentary in lateral view ([17]: character 41, http://www.morphbank.net/Show/?id=461244) is variable within the sample. The bulging is just rostral to the coronoid process in MCD-4945 and possibly MCD-5096; it occurs below the coronoid process in MCD-5008; the margin is straight in MCD-5007 and MCD-4743. The coronoid process appears slightly inclined rostrally with respect to the main axis of the dentary battery in MCD-4744, MCD-4945, MCD-5096, MCD-5097, MCD-5007 and possibly also in MCD-5008, the angle ranging from 74.5º (MCD-5096) to 76º (MCD-5007). A slightly higher inclination (~80º) seems to have occurred in MCD-5012 (this was observed in the field, where the impression of the coronoid process was still visible; see Fig 3M). The coronoid process is practically vertical in MCD-4946 (85°), which is comparable in size with MCD-4945. The extent of this sloping has been considered to be of taxonomic significance ([17]: character 42), with an angle greater than 82° occurring in non-hadrosaurid hadrosauroids and an angle between 69° and 82° occurring in most hadrosaurids. We measured this angle with respect to the main axis of the dentary battery (keeping the main axis [= elongation axis] of the elliptical dental battery horizontal), whereas Prieto-Márquez [17] measured it with respect to the dorsal margin of the alveolar sulci of the dental battery. We did this because the dorsal margin of the alveolar sulci is always more or less damaged and irregular in the BP specimens (Fig 3). The variability of the angle in the BP sample probably is a consequence of the biasing factors noted above. Furthermore, it could reflect intraspecific (ontogenetic and individual) variability, as with other morphological features in the BP sample. This is suggested by the lack of a neat separation into two groups among the BP dentaries.

The tooth family count, based both on the count of preserved dental sulci in toothless specimens and in situ teeth, and the estimate of the missing ones (no one battery is complete), ranges from 25–28 in small dentaries (MCD-4743, MCD-4744, MCD-4945, MCD-4963, MCD-5008, MCD-5012 and MCD-5096) to 33–34 in the larger dentary MCD-5007. The count in the small dentaries lies within the range of the non-hadrosaurid hadrosauroids ([3]: SI), but this has no phylogenetic implications unless they belong to adult individuals because the number of tooth positions increased with age (e.g. [44]). The MCD-5007 count is within the range of most hadrosaurids (31–42; [3]: SI) and closer to lambeosaurines such as *Charonosaurus jiayinensis*, *Corythosaurus intermedius*, *Blasisaurus canudoi* and to the dentary of an indeterminate lambeosaurine that formerly was the holotype of *Koutalisaurus kohlerorum* (see [3,45]).

The position of the caudal end of the dental battery is variable within the BP sample. It is found flush with the caudal margin of the coronoid process (specimens MCD-4744, MCD-4945, MCD-4963 and MCD-5008), or possibly slightly caudal to it (MCD-5012, MCD-5007 and MCD-4743). It is slightly rostral to the caudal margin of the coronoid process in MCD-5096; and it is rostral to the caudal margin of the process in MCD-4946. The sample thus presents all three of the character states coded by Prieto-Márquez [17], suggesting that this character is not diagnostic or that its state in the BP sample is biased by taphonomic factors. The former possibility has important implications, since the position of the caudal end of the dental battery has been widely considered in hadrosauroid phylogenetic analyses (e.g. [17,35,46].

The dental sulci are narrow and parallel-sided; they are straight in MCD-4836, 4945, 4963, 5007 and 5008, but their ventral part is curved rostrally in MCD-4946.

**Fig 4:**
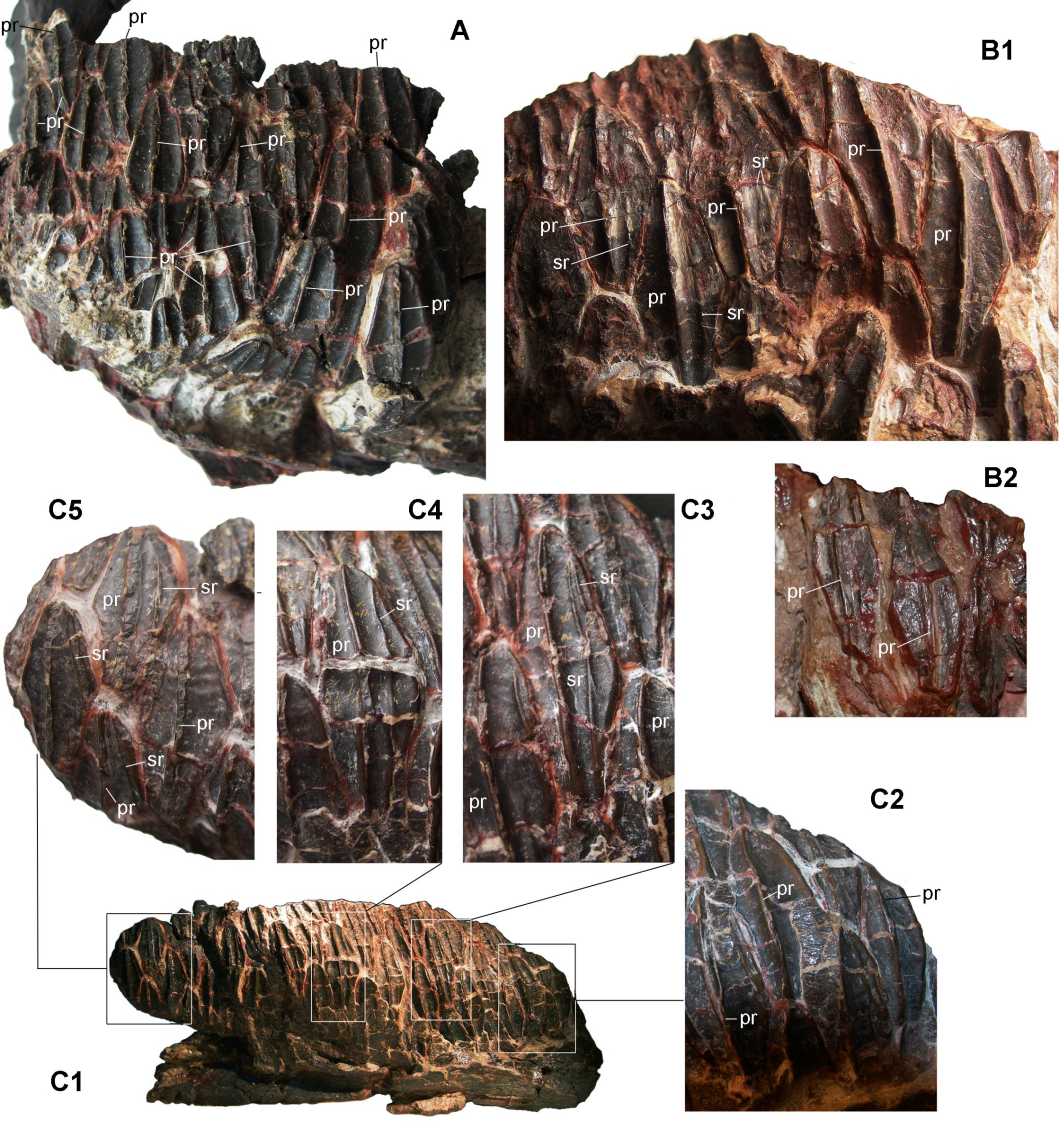
Dentary tooth variability in three small BP dentaries (in lingual view). (A) MCD-5096 (caudal half of the tooth battery). (B) MCD-4743, distal (B1) and centro-proximal teeth (B2). (C) MCD-5012, the whole tooth battery (C1), mesial (C2), mesio-central (C3), centro-distal (C4) and distal (C5) teeth. Abbreviations: pr, primary ridge; sr, supplementary (accessory) ridge. For the whole MCD-5096 and MCD-4743 specimens and scale, see Fig 3.

Teeth are partly preserved in situ only in three small-sized specimens (MCD-4743, MCD-5096 and MCD-5012; Fig 3K, 3L and 3Q). MCD-4743 and MCD-5096 are similar in size (estimated total length 190 mm), whereas MCD-5012 is slightly larger (estimated total length ~220 mm), but all of them fall within the small size-class (see below). Only the distal half of the tooth battery is preserved in MCD-5096 (Figs 3K and 4A), whereas MCD-4743 preserves the distal third and a portion of the mid-mesial part of the battery (Figs 3Q and 4B). MCD-5012 preserves most of the tooth battery (Figs 3L and 4C). The BP sample also includes the distal-most portion of a dental battery with teeth from a larger right dentary (MCD-4726a), probably belonging to the large size-class (see below).

In these specimens, three teeth are present per tooth family in lingual view. Three teeth per tooth family at mid-length of the dental battery occur in non-hadrosaurid hadrosauroids, but also in many lambeosaurines, including *Arenysaurus ardevoli* and *Blasisaurus canudoi* (see [3]: character 2, SI).

In the dental battery of the BP dentaries, there are three functional teeth per family exposed on the dentary occlusal plane. MCD-4726a has only two functional teeth, but it preserves only the distal-most teeth. Three functional teeth throughout most of the dental battery, decreasing to two near the rostral and caudal ends of the dentary battery, is the usual condition within hadrosaurids ([3]: character 3(2), SI).

In MCD-5012, the basoapical height/width ratio of the tooth crowns ranges from 2.6–2.9 in the mesial teeth, 2.8–2.9 in the mid-battery teeth and 2.5 in the distal teeth (some mesial crowns are broader than the mid-battery crowns). In MCD-5096, this ratio is 2.7–2.9 in the mid-battery teeth and 2.5–2.9 in the distal teeth. In MCD-4743, the ratio is 2.6–2.7 in the mid-mesial teeth and 2.3–2.5 in the mid-distal teeth. The larger MCD-4726a has more slender teeth with a height/width ratio of 3.0–3.1. This latter ratio is shared with some non-hadrosaurid hadrosauroids, saurolophine hadrosaurids and also lambeosaurine hadrosaurids ([3]: character 4, SI). The comparatively low height/width ratio of the crowns of smaller dentaries is shared by some non-hadrosaurid hadrosauroids, *Hadrosaurus foulkii* and some saurolophine hadrosaurids ([3]: character 4, SI; [17], http://www.morphbank.net/Show/?id=461204; [41]), but this could be due to an early ontogenetic stage of the small BP dentaries (see below) rather than to a taxonomic affinity.

The ridging pattern varies considerably among the teeth of MCD-4743, MCD-5096 and MCD-5012 (Fig 4). MCD-5012 (Fig 4C1) has a relatively complex pattern. There is a thin and scarcely prominent, median primary ridge and a thinner mesial, supplementary (accessory) ridge (Fig 4C2–4C5). The primary ridge appears to be slightly sigmoid in the mid-mesial crowns because it bends mesially at its apical end; this apical bending seems to be lacking in the mid-distal teeth, whereas the ridge of distal teeth is curved distally. The supplementary ridge is rather variable in length and position. In some teeth, it spans the whole crown; sometimes another, short mesial ridge starting from the mesial edge is confluent with it in the upper half of the crown or is parallel with it (Fig 4C3); occasionally, only this latter shorter ridge occurs. Sometimes, the supplementary ridge is centred in the lower half of the crown and there is a distinct supplementary ridge in the upper half of the crown very close to the thick mesial margin (Fig 4C3). One tooth has two faint and very short additional ridges in the upper part of the crown (thus, it has a total of four supplementary ridges). A supplementary ridge is lacking in a few mesial-most crowns (Fig 4C2) and in one distal crown (Fig 4C5). The crowns of MCD-5096 have only a single, median primary ridge that is thin and scarcely prominent (Fig 4A); the ridge is straight in the median teeth and slightly curved distally in at least some distal teeth. MCD-4743 (Fig 4B) has a ridging pattern that is intermediate between those of MCD-5012 and MCD-5096. A single median primary ridge that is thin and low occurs in the mid-mesial teeth and in the mesial teeth of the mid-distal preserved portion of the dentition (Fig 4B1 and 4B2), as in MCD-5096, but a thinner, mesial, supplementary ridge is visible in a few distal crowns (Fig 4B1). The apical end of the median ridge is slightly bent mesially in one mid-mesial tooth; it is slightly bent distally in the mid-distal teeth, following the backward bending of the crown. The primary ridge always is offset slightly at the distal in all three specimens. This variability in ridging pattern in small-sized dentaries is concordant with intraspecific (individual) variability, rather than with taxonomic distinctions. The distal teeth of the larger MCD-4726a have a single, median primary ridge that is thin and sigmoid. Apically, the ridge curves following the distal bending of the crown, but it curves mesially at the apex in the last lower replacement crown; the ridge is also slightly displaced distally in this crown. The presence of supplementary ridges in dentary crowns is a primitive feature of non-hadrosaurid hadrosauroids, but a supplementary mesial ridge also occurs in the dentary crowns of lambeosaurines [3,17,35,47]. The supplementary ridge pattern in lambeosaurines is highly variable, and teeth of the same tooth battery can have or lack supplemetary ridges [3].

Both the mesial and the distal margins of the apical half of the tooth crowns bear small, papilla-like denticles in the BP specimens; the arrangement and fine morphology of these denticles is variable among the specimens and within a single dentition. The marginal denticles of non-hadrosaurid hadrosauroids such as *Telmatosaurus transsylvanicus* and *Tethyshadros insularis* are wedge to tongue-shaped ([3]: character 15, SI, http://www.morphbank.net/Show/?id=461223; [41]).

It is impossible to identify two or more groups of dentaries in the BP sample, each of which is clearly distinct morphologically. All of the specimens are roughly similar, and all of them differ from one another in minor details that are plausibly caused by taphonomic processes or are due to intraspecific variability. The only apparent exception is MCD-4946, which differs in the less inclined coronoid process, the posterior termination of the tooth battery that is rostral to the caudal margin of the coronoid process, and the mesially curved ventral part of the alveolar sulci. However, this could be the result of deformation caused by taphonomic processes as well as by an unfaithful restoration of the specimen (the coronoid process was glued to the body of the dentary).

**Fig 5:**
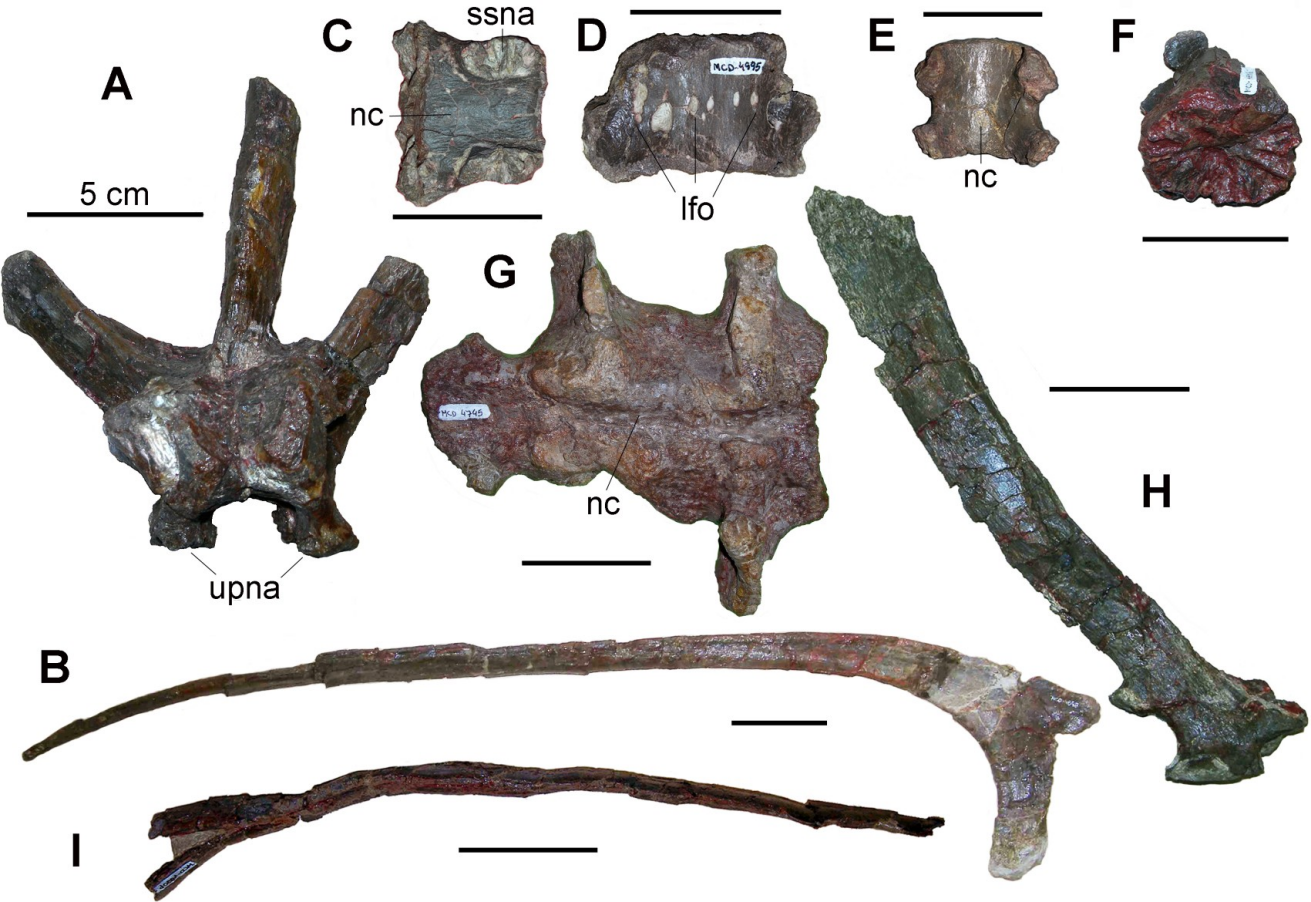
Axial elements from Basturs Poble bonebed. (A) MCD-4891, neural arch of a dorsal vertebra, anterior view. (B) MCD-4848, dorsal rib, anteroposterior view. (C) MCD-4966, isolated sacral centrum, dorsal view. (D) MCD-4995, isolated sacral centrum, ventral view. (E) MCD-4897, isolated sacral centrum, dorsal view. (F) MCD-4851b, isolated sacral centrum, articular view. (G) MCD-4745, fragment of a synsacrum, dorsal view. (H) MCD-4938, neural arch of a proximal caudal vertebra, right lateral view. (I) MCD- 4850, proximal chevron, anteroposterior view (horizontal, instead of its natural vertical orientation). Abbreviations: lfo, large foramina; nc, neural canal; ssna, sutural surface for the neural arch; upna, base of the unfused pedicels of the neural arch. Scale bar equals 5 cm.

Axial elements—Axial bones (including the few collected ossified tendons) account for 44% of the sample. The chevrons from the BP sample have long, strap-like spines. The largest and most complete specimen (MCD-4850, Fig 5I), probably from the beginning of the chevron series, has an extremely elongated and caudocranially narrow spine and represents an unusually deep proximal part of the tail. In hadrosaurids, chevrons are longer than the corresponding neural spines, or the two are equal in proportions [35]. Proximal caudal vertebrae with chevrons longer than the neural spines constitute an ambiguous synapomorphy of the Saurolophinae for Prieto-Márquez [17]. The most complete neural spines of proximal caudal vertebrae from BP bonebed are also slender and elongated, but proportionally less so than MCD-4850 (Fig 5H). Lambeosaurines have comparatively taller proximal caudal neural spines with respect to most basal hadrosauroids [35]. The non-hadrosaurid hadrosauroids from the upper Campanian-lower Maastrichtian of the European Archipelago, *Telmatosaurus transsylvanicus* and *Tethyshadros insularis*, have much lower and broader neural spines and comparatively shorter chevrons than those in the proximal caudal vertebrae of the BP sample [41,48]. Weishampel et al. [49] considered very tall neural spines apomorphic for Lambeosaurinae.

**Fig 6:**
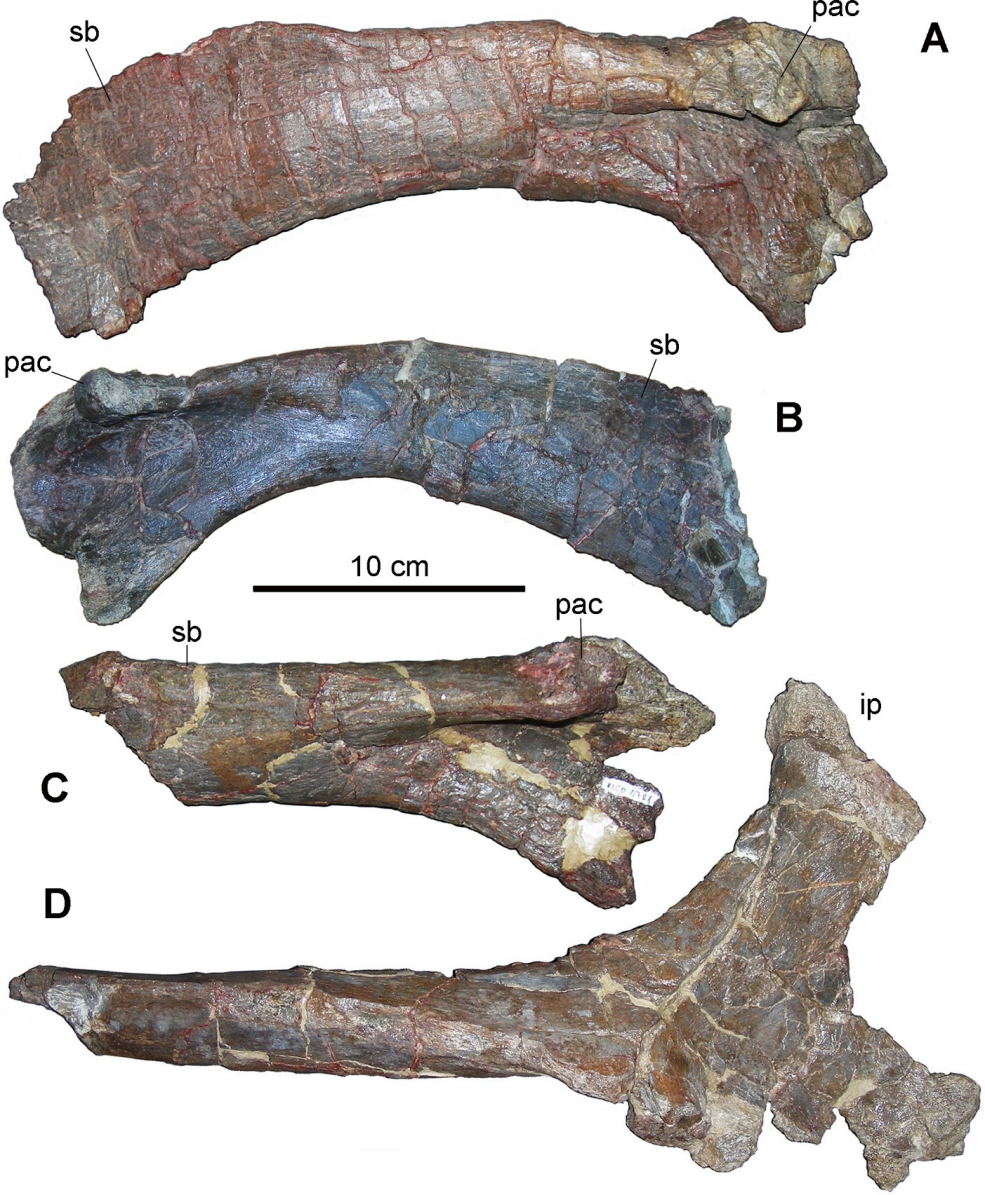
Elements of the girdles from Basturs Poble bonebed. (A) MCD-4738, right scapula. (B) MCD-4826, left scapula. (C) MCD-4781, right scapula. (D) MCD-4881, proximal portion of a right ischium. Abbreviations: ip, iliac peduncle; pac, pseudoacromion process; sb, scapular blade. Scale bar equals 10 cm.

Girdles—Elements from the girdles are under-represented. The lateral profile of the dorsal margin of the scapula is craniocaudally straight in MCD-4738 and MCD-4781 (Fig 6C), whereas it is curved and dorsally convex in MCD-4826 (Fig 6B). In MCD-4738, the degree of curvature is intermediate between that of MCD-4781 and 4826 (Fig 6A). Straight and curved scapulae were considered to be characteristic of distinct clades by Prieto-Márquez [17], but the gradual transition between the two extremes suggests that curvature is intraspecifically variable in the BP sample. The ratio between the dorsoventral width of the proximal constriction (“neck”) and the dorsoventral depth of the cranial end of the scapula ([17]: character 214, http://www.morphbank.net/Show/?id=461717) is 0.51 in MCD-4781, 0.56 in MCD-4738, and 0.55 in MCD-4826. The “neck” is thus narrow (ratio ≤0.60) in all these specimens, as it is in many hadrosauroids, including some lambeosaurines (e.g., *Arenysaurus ardevoli*; [3]: SI). The scapula IPS-693-3, referred to *Pararhabdodon isonensis* (see [50]), also has a quite narrow “neck”. However, the measurements on which this ratio is based could be affected by preservational factors. Scapulae MCD-4826 and MCD-4781 have a rostrodorsally directed pseudoacromion process [14], a feature that is common within the Lambeosaurinae [17]. The deltoid ridge is dorsoventrally deep and craniocaudally long, with a relatively well-demarcated ventral margin (character state 1 of character 218 in [17]) in comparison to the examples for character state 0 and 1 of character 218 in Prieto-Márquez ([17], http://www.morphbank.net/Show/?id=461720). However, the deltoid ridge is demarcated more clearly in the larger MCD-4717 than in the other BP specimens, and it is much fainter in MCD-4738 (Fig 6A) than in the example for character state 218–1 in Prieto-Márquez ([17], http://www.morphbank.net/Show/?id=461720). According to Brett-Surman and Wagner [51], the deltoid ridge is more clearly demarcated in adults than in juveniles; this would suggest that the scapulae from BP bonebed are all from non-adults. However, the proximal part of a left scapula (IPS-693-3) referred to *P*. *isonensis* (see [50]) has a very faint deltoid ridge, although it is much larger than the largest BP specimen.

No ilia and pubes were found. The curvature in the caudodorsal corner of the distal margin of the iliac peduncle of the large ischium MCD-4881 (Fig 6D) is well-developed in lateromedial view (“thumb-like”). This is a synapomorphy of the Lambeosaurinae [3, 17].

**Fig 7:**
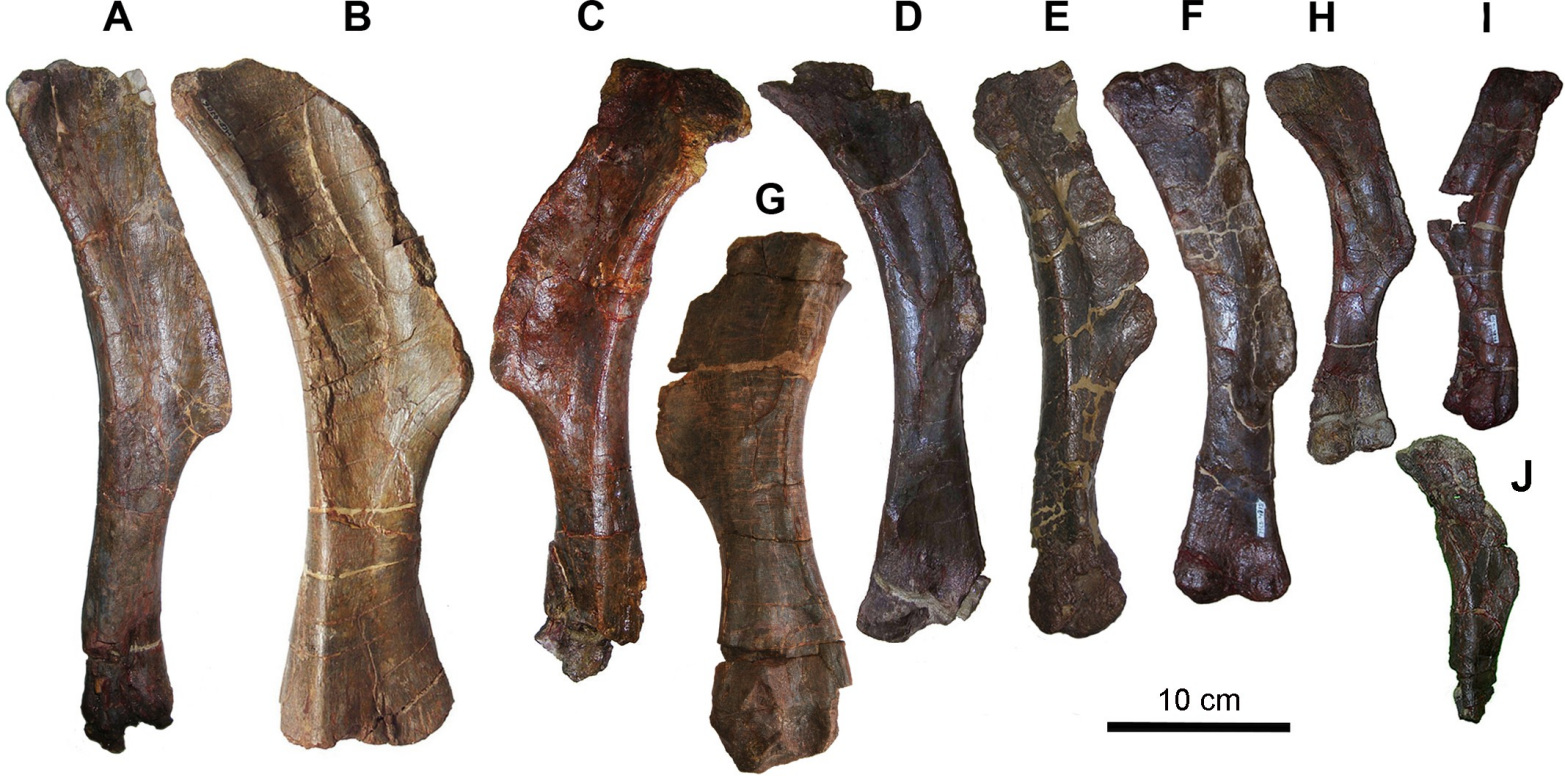
Humeri from Basturs Poble bonebed. (A) MCD-4825, left. (B) MCD-4706, left. (C) MCD-4845, right. (D) MCD-5021, left. (E) MCD-4746, left. (F) MCD-4818, left. (G) MCD-5376a, left. (H) MCD-5009, left. (I) MCD-4846, right. (J) MCD-5110, left. Scale bar equals 10 cm.

Limb elements–Ten bones are sufficiently complete to be identified as humeri. According to the overall proportions of the humerus measured as a ratio between the total length and the width of the lateral surface of the proximal end ([17]: character 222, http://www.morphbank.net/Show/?id=461724), the sample includes a stocky and a slender morph. The former (MCD-5021, MCD-4818, MCD-5009 and MCD-4846, Fig 7D, 7H and 7I) has a ratio ranging from 3.80–3.97 independently of size, which is within the range for lambeosaurines, whereas the latter (MCD-4825, Fig 7A; ratio = 5.0) lies within the range of some saurolophines. MCD-4706 (Fig 7B) and MCD-5376a (Fig 7G) are also evidently stockier than MCD-4825, whereas MCD-4845 (Fig 7C) is intermediate between the two. MCD-4825 and 4706 have similar total length, but the width just proximal to the distal condyles is 52 mm and 94 mm, respectively. The outline of the deltopectoral crest (DPC) appears to be variable in the sample, but the humeri cannot be grouped into two or more distinct sets based on this feature (Fig 7). The DPC of MCD-4825 (Fig 7A) has a distinct angular (nose-like) profile, whereas that of MCD-5021 (Fig 7D) is rounded and that of MCD-4818 (Fig 7F) is intermediate in shape. The DPC of the smaller MCD-5009 (Fig 7F) is as rounded as that of MCD-5021, but it is more prominent. The DPC width/total length ratio is variable and ranges from 0.15 (MCD-4746) to 0.23 (MCD-4845). The DPC extends along more than half the total length of the humerus: the DPC length/total length ratio ranges from 0.52 (MCD-4746) to 0.57 (MCD-4818). All saurolophids are within the range of the BP sample, whereas non-hadrosaurid hadrosauroids have a DPC length/total length ratio lower than 0.48 ([3]: character 202, SI, http://www.morphbank.net/Show/?id=461721). The DPC is oriented anterolaterally in most specimens, but appears to be oriented anteriorly in specimens MCD-4746, MCD-5021 and MCD-4846. However, the DPC orientation appears to be biased by crushing, and its taxonomic significance must be evaluated with caution. The BP humeri differ from those of non-hadrosaurid hadrosauroids in the length and angulation of the DPC [41,49].

**Fig 8:**
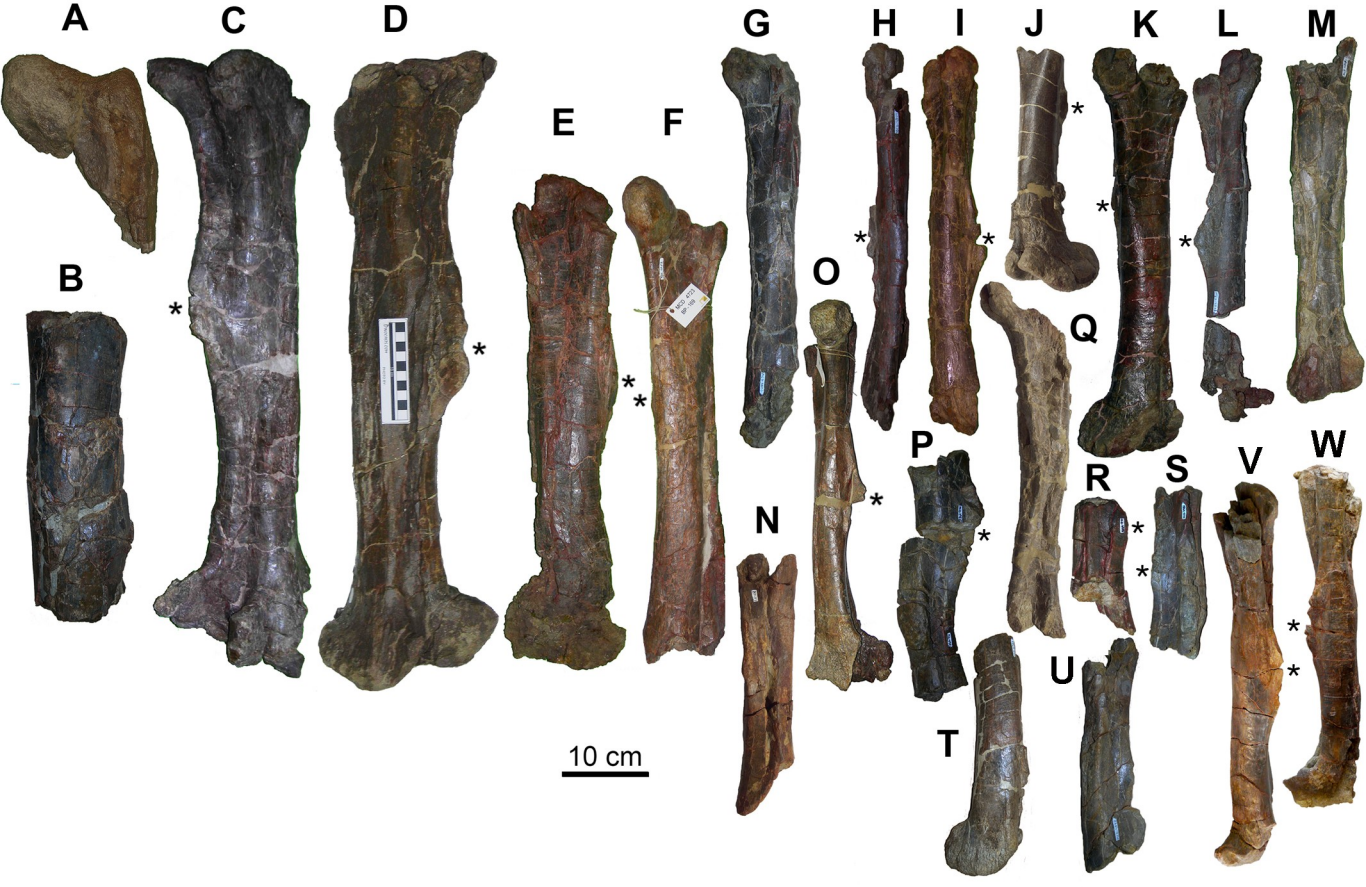
Femora from Basturs Poble bonebed, in approximate order of decreasing size. (A) MCD-4742, right. (B) MCD-4941, left. (C) MCD-5107, right. (D) MCD-5011, left. (E) MCD-4754, right. (F) MCD-4723, left. (G) MCD-4800, left. (H) MCD-4804, right. (I) MCD-4801, right. (J) MCD-4708, right. (K) MCD-4729, left. (L) MCD-4802, left. (M) MCD-4704, left. (N) MCD-5104, right. (O) MCD-4702, right. (P) MCD-4987, left. (Q) MCD-4722, right. (R) MCD-4998, left. (S) MCD-4983, right. (T) MCD-4783, left. (U) MCD-4892, right. (V) MCD-5369, right. (W) MCD-5370, left. The asterisk indicates the position of the 4th trochanter. Scale bar equals 10 cm.

**Fig 9:**
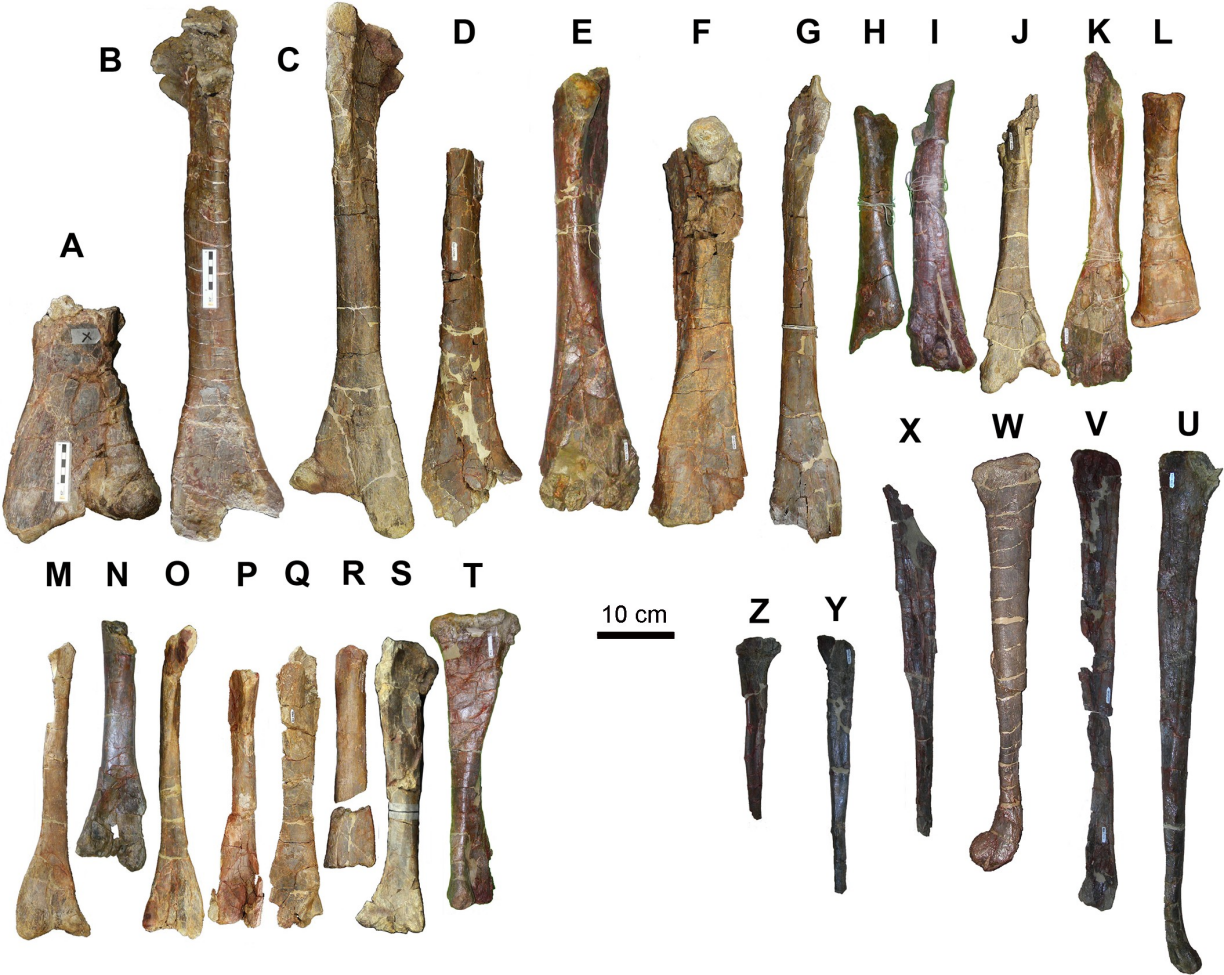
Tibiae and fibulae from Basturs Poble bonebed, in approximate order of decreasing size. Tibiae: (A) MCD-5109, right. (B) MCD-4728, right. (C) MCD-4958, left. (D) MCD-4918, right. (E) MCD-4701, right. (F) MCD-4920, left. (G) MCD-4719, left. (H) MCD-5105, right. (I) MCD-4705, left. (J) MCD-4784, right. (K) MCD-4771, right. (L) MCD-4886, left. (M) MCD-4796, right. (N) MCD-4986, left. (O) MCD-4799, left. (P) MCD-4882, right. (Q) MCD-4956, right. (R) MCD-4721, right. (S) MCD-7144, left. (T) MCD-5106, left. Fibulae: (U) MCD-4889, left. (V) MCD-4912, right. (W) MCD-4715, left. (X) MCD-4795, right. (Y) MCD-4741, right. (Z) MCD-4985, left. Scale bar equals 10 cm.

Femora (23 specimens; Fig 8) and tibiae (20 specimens; Fig 9) are the most common appendicular elements in the BP sample; these were used for the histological study. The position of the fourth trochanter along the femur shaft appears to be quite variable in the BP sample. This trochanter is located in the middle of the femur (MCD-4801 and MCD-5011; Fig 8D and 8I), or it extends more on the upper half (MCD-4729, MCD-4754 and MCD-5107; Fig 8C, 8E and 8K) or more on the lower half (MCD-4702 and MCD-4708; Fig 8J and 8O) of the shaft. Although damaged in all specimens, the fourth trochanter also seems to be variable in morphology, always being triangular, but with a variable degree of asymmetry (it is markedly asymmetrical in MCD-4702 and MCD-4983, whereas it is symmetrical in MCD-4801). The fourth trochanter is also rather variable in extension along the shaft. The cranial intercondylar groove of the femur is open in MCD-4708, whereas it is closed (tunnel-like) in MCD-5011.

As with the humeri, some tibiae are more robust than others of similar size (e.g., compare MCD-4886 and MCD-4986 with MCD-4796; Fig 9L, 9M and 9N).

The cranially expanded distal end of the fibula is club-shaped in MCD-4715 (Fig 9W) and MCD-4889 (Fig 9U), with a prominent cranial/anterior expansion. This cranial/anterior expansion is a lambeosaurine feature that is convergent in *Tanius sinensis* and reversed in the *Hypacrosaurus altispinus*–*Amurosaurus* clade [17]. As with the tibiae, the fibulae vary in robustness: MCD-4715 is more robust than MCD-4889 (compare Fig 9W and 9U).

In short, there is great but gradual variability in morphology among some skeletal elements in the sample, which is attributable to intraspecific variability rather than to taxonomical distinctions. The presence of two morphs that differ in robustness is evident among the humeri, tibiae and fibulae. The taxonomically significant features in the sample (features of the maxilla, jugal, quadrate, frontal, humeri, ischium and fibulae) suggest that the BP hadrosauroids belong to the Lambeosaurinae clade, both in the case of small-sized and medium to large-sized individuals (see below). Primitive features shared with non-hadrosaurid hadrosauroids also occur within the Lambeosaurinae, or are sporadically present in the sample and ascribable to individual variability or taphonomic biases.

### Size and features of immaturity

Among the 50 tibiae belonging to the saurolophine *Maiasaura peeblesorum* from a single upper Campanian bonebed in the USA, individuals 4–15 years old according to histological studies have tibiae ranging from 720–980 mm in length, and skeletal maturity was postulated at age eight years [19]. In a sample of 56 hadrosaurid femora and tibiae from the upper Campanian Dinosaur Park Formation of Alberta (Canada), the lengths of femora and tibiae of individuals belonging to the adult size-class range from 785–1144 mm and from 710–1034 mm, respectively, with a peak in the range from 993–1040 mm for femora [52]. These two samples provide a reference for the body size of adult hadrosaurids.

Cranial elements—The few cranial elements from the BP bonebed belong to small-sized individuals. The estimated total length of the most complete maxilla (MCD-5099; Fig 2A) is 110–115 mm. As the maxillary tooth row (used as a proxy for maxilla size) ranges from 260–436 mm in length in a sample of presumed adult North American hadrosaurids (the saurolophines *Edmontosaurus regalis*, *E*. *annectens*, *Kritosaurus navajovius*, *Prosaurolophus maximus* and *Saurolophus osborni*; and the lambeosaurines *Corythosaurus casuarius*, *Lambeosaurus lambei* and *Hypacrosaurus altispinus*; [53]), MCD-5099 belongs to a rather small-sized individual by hadrosaurid standards.

The nearly complete jugal (MCD-5100; Fig 2B) is 95 mm long; the frontal (MCD-4869a; Fig 2C) is 53 mm long, and the complete right exoccipital (MCD-4851a; Fig 2E) is 44.5 mm tall and 48 mm wide. Taking as a reference the reconstruction of the skull of the lambeosaurine *Tsintaosaurus spinorhinus* in Prieto-Márquez and Wagner [39] for scaling, the jugal MCD-5100 would correspond to a skull 385 mm long. The skull length in a sample of presumed adult North American hadrosaurids (femur length≥1 m) ranges from 720–1200 mm (the skull is proportionally shorter in lambeosaurines; [53]). That sample includes the saurolophines *Edmontosaurus regalis*, *E*. *annectens*, *Gryposaurus notabilis*, *Prosaurolopus maximus* and *Saurolophus osborni*; and the lambeosaurines *Corythosaurus casuarius*, *Lambeosaurus lambei*, *Hypacrosaurus altispinus* and *Parasaurolophus walkeri*. The ratio skull length/femur length is 1.04 in *S*. *osborni*, 0.90 in *E*. *regalis* and *P*. *maximus*, 0.85 in *G*. *notabilis*, 0.78 in *P*. *walkeri*, 0.77 in *L*. *lambei*, 0.70 in *C*. *casuarius* and 0.67 in *H*. *altispinus*.

The right quadrate MCD-5278 (Fig 2D) is 198 mm tall (measured along the straight line uniting the distal and proximal condyles). It belongs to an individual that is larger than those represented by the other skull bones. Taking as a reference the reconstruction of the skull of *Tsintaosaurus spinorhinus* (nearly one metre long) in Prieto-Márquez and Wagner [39] for scaling, the BP quadrate would correspond to a skull about 520 mm long. Quadrates in a sample of presumed adult North American hadrosaurids (femur length>1 m) range from 280–445 mm in length [53]. This sample includes the saurolophines *Edmontosaurus regalis*, *E*. *annectens*, *Gryposaurus notabilis*, *Prosaurolophus maximus* and *Saurolophus osborni*; and the lambeosaurines *Corythosaurus casuarius*, *C*. *intermedius*, *Lambeosaurus lambei*, *Hypacrosaurus altispinus* and *Parasaurolophus walkeri*. Therefore, MCD-5278 belongs to an individual of comparatively modest size by hadrosaur standards.

**Fig 10:**
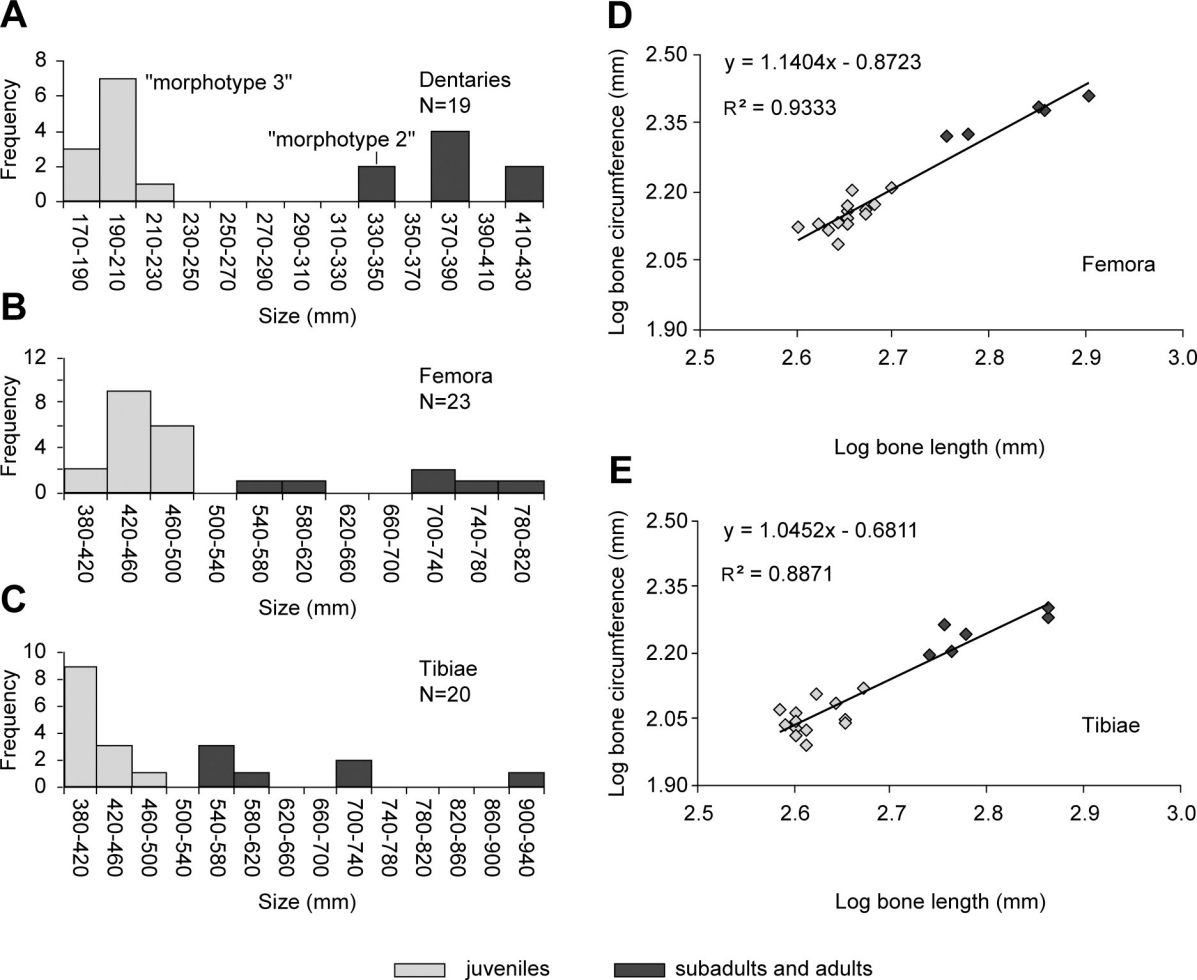
Size (length) distribution of BP dentaries, femora and tibiae. (A) Length of dentaries. (B) Length of femora. (C) Length of tibiae. (D) and (E) Scatter-plots of tibiae and femora, respectively. Distinction between juveniles and adult/subadults is based on the histological information. The "morphotype 2" and "morphotype 3" of Blanco et al. [15] are indicated in A. For the measurements, see Tables A-C in the S1 Text.

Dentaries—The nine most complete dentaries are small-sized, apart from MCD-5007 (Fig 3C); the larger dentaries are very fragmentary. The length of the most complete dentaries as preserved ranges from 120 mm to 285 mm, with estimated total length ranging from 170 mm (MCD-4963) to 340 mm (MCD-5007). The very fragmentary MCD-4779 (Fig 3A) might represent the largest dentary in the sample. Apart from MCD-5007, the estimated lengths ranging from 330–430 mm of Fig 10A are just indicative. There are two size-classes (Fig 10A), one of small individuals (170 < length < 230 mm) and one of larger individuals (330 < length < 430 mm) represented mostly by very fragmentary remains. Comparison with the size distribution of postcranial remains (e.g., femora and tibiae; Fig 10A) shows that this subdivision into only two size-classes is taphonomically biased. In large and presumed adult individuals belonging to various hadrosaurid species (both saurolophine and lambeosaurine) from western North America and Asia (femur length >1000 mm), the dentary lengths range from 450–900 mm [34,53,54]. Thus, the largest dentaries in the BP sample are of modest size by hadrosaurid standards.

Axial elements—The total length of the opisthocoelous centra of the cervical vertebrae ranges from 50–65 mm. Dorsal vertebrae are represented only by partial neural arches and spines. The pedicels of the neural arch MCD-4891 (Fig 5A) that is from a proximal dorsal vertebra were not fused to the centrum, as indicated by the rough articular facets of the pedicels, despite the relatively large size of the vertebra. The most complete dorsal ribs, MCD-4848 (Fig 5B) and MCD-4865, are 573 mm and 460 mm long, respectively (measured as the straight line between the two extremities). In addition to their small size, most of the sacral vertebrae have dorsoventrally flattened centra and broad neural canals (Fig 5C–5F); the latter are characteristic of juvenile individuals [35]. Some isolated and very small centra (length ranging from 40–41.5 mm; Fig 5C–5F) have rough and radially grooved articular surfaces, showing that they were closely apposed but not fused to adjacent centra (Fig 5F). Their neural arches were also unfused (Fig 5C). Most isolated centra have very large nutritive foramina on the lateroventral sides, sometimes organized in rows (Fig 5D). The three partial synsacra belong to larger individuals than those represented by most unfused centra (MCD-4745 is 147 mm long and is formed by three fused centra; Fig 5G). The holotype of the saurolophine *Gryposaurus incurvimanus*, with a 1045-mm-long femur, has a sacrum consisting of nine fused vertebrae that is at least 570 mm long [55]; the eight fused vertebrae of the sacrum of the lambeosaurine *Pararhabdodon isonensis* (see [50]) are about 550 mm long. Caudal vertebrae are the most common vertebrae, in agreement with their higher number in the hadrosaurid skeleton (total 50–70, with 12–15, 16–20 and 9–12 vertebrae of the neck, torso and sacrum, respectively; [35]). All of these caudal vertebrae are modest in size, the lengths of the centra ranging from 25.3–65.5 mm, with 80% of the centra < 45 mm long. Evidence of immaturity is widespread among the caudal vertebrae. Most of the incomplete and isolated neural arches in the sample were probably unfused to their centra. In some specimens, the neurocentral suture remains partly visible. However, some relatively small caudal vertebrae do have fused arches, while other larger specimens do not. The most complete and largest chevron (MCD-4850; Fig 5I) is a proximal element over 600 mm long. The longest chevron from specimen AMNH 5240 of the relatively deep-tailed *Corythosaurus casuarius*, with a femur 1080 mm long, is about 370 mm length [56]. Thus, MCD-4850 undoubtedly belongs to a large-sized individual, and it is unusually elongated as well.

Girdles—The girdle elements belong to very small and much larger individuals, although the latter actually are not very large by hadrosaurid standards. The maximum dorsoventral length of the only coracoid is 107 mm. In the holotype of the saurolophine *Gryposaurus incurvimanus*, with a 1045-mm-long femur, the coracoid is 200 mm long [55]. The most complete scapulae are modest in size by hadrosaurid standards (the length is about 300–350 mm, but the scapular blade is incomplete distally; see Fig 6). In large and presumed adult individuals belonging to hadrosaurid species (both saurolophine and lambeosaurine) from western North America (femur length >1000 mm), the scapula length ranges from 776–940 mm [53]. The BP sample includes the proximal part of three ischia. MCD-4881 (Fig 6D) is much larger than the others and is one of the largest skeletal elements in the BP sample.

Limb elements–The total lengths of humeri range from 215 mm to 425 mm (the smallest value was estimated) (Fig 7A). In large and presumed adult individuals belonging to hadrosaurid species (both saurolophine and lambeosaurine) from western North America (femur length >1000 mm), humerus length ranges from 501–694 mm [53]. Only two ulnae (MCD-4724 and MCD-4725, 405 mm and 204 mm long, respectively) are nearly complete, whereas the others are very fragmentary. The largest of the latter (MCD-4841) is a distal half that is 275 mm long. The only nearly complete radius (MCD-4752) is 415 mm long. In the western North American hadrosaurid sample mentioned above, the ulna ranges from 548–680 mm in length [53].

The size distribution of femora and tibiae (Fig 10B and 10C) can be compared directly with the size distributions reported in Woodward et al. [19] and Brinkman [52]. For tibiae and femora, over-representation of small individuals and presence of distinct size-classes observed in the skull, lower jaw and axial bones can be quantified. There seem to be three size-classes for femora (Fig 10A and 10C). The small size-class (length from 400 mm to 490 mm) accounts for most of the specimens (76%); the medium size-class (estimated length from 550 mm to 600 mm) consists of only two specimens (MCD-4723 and MCD-4754; Fig 8E and 8F); the large size-class includes two femora (MCD-5107 and MCD-5011; Fig 8C and 8D) with lengths of 710 mm and 720 mm, respectively. A shaft segment (MCD-4941; Fig 8B) belongs to a slightly larger femur, possibly up to 800 mm in length.

There seem to be three or four size-classes for tibiae (Fig 10A and 10B). The small size-class (length from 390 mm to 450 mm) accounts for most of the specimens (61%); the medium size-class (length from 550 mm to 600 mm) consists of only four specimens (MCD-4918, MCD-4701, MCD-4920 and MCD-4719; Fig 8D–8G); the large size-class includes two complete tibiae (MCD-4728, right, and MCD-4958, left; Fig 8B and 8C) that are 730 mm long. MCD-5109 (Fig 8A) is the distal portion of a right tibia with estimated total length of 940 mm (Table C in S1 Text) and which possibly represents a fourth size-class.

The histograms of tibiae, femora and dentaries (Fig 10A–10C) are right-skewed, indicating higher mortality among the smallest individuals. This pattern resembles the histograms of the tibiae from the *Maiasaura peeblesorum* bonebed [19], although the absolute lengths of the tibiae in the BP sample are smaller than in the *M*. *peeblesorum* sample. In fact, only one tibia, representing 6% of the sample, is longer than 740 mm in the BP sample, whereas 24% are over 740 mm long in the *M*. *peeblesorum* sample.

The two complete fibulae (MCD-4889 and MCD-4715; Fig 9U and 9W) are 680 mm and 550 mm long, respectively. As the fibula is only slightly shorter than the tibia in hadrosauroid dinosaurs, MCD-4715 belongs to the medium size-class and MCD-4889 to the large size-class.

Metatarsals II, III, IV from BP range from 106–210 mm, 105–272 mm, and 82.5–217 mm in length, respectively. Metatarsals III from the above-mentioned North American sample (the saurolophines *Edmontosaurus regalis*, *E*. *annectens*, *Gryposaurus incurvimanus*, *Parasaurolophus maximus* and *Saurolophus osborni*; and the lambeosaurines *Corythosaurus casuarius*, *Lambeosaurus lambei* and *Hypacrosaurus altispinus*) range from 320–420 mm in length.

We conclude that the sample represents a wide size range, with three or possibly four size-classes. The small size-class is over-represented, but very small individuals (hatchlings) are absent and the largest individuals from BP are as large as the small adults of North American hadrosaur taxa [52].

## Bone histology and ontogenetic stages

Tibiae were preferred to femora for histological analyses, following Woodward et al. [19], because of possible interferences with regional tissue growth related to the development of the fourth trochanter (which extends to nearly half the length of the diaphysis in hadrosauroid femora). We selected nine tibiae (Fig A in S2 Text), representing nine different individuals, on the basis of the following criteria: (1) the bones were taken from the right side; (2) left tibiae were also selected when they differed in length from all of the right tibiae by at least ≈10%. In order to test the results of the histological observations on the tibiae, five femora were also selected for histological analysis using the same criteria (Fig A in S2 Text).

### Description of bone histology and interpretation

**Fig 11:**
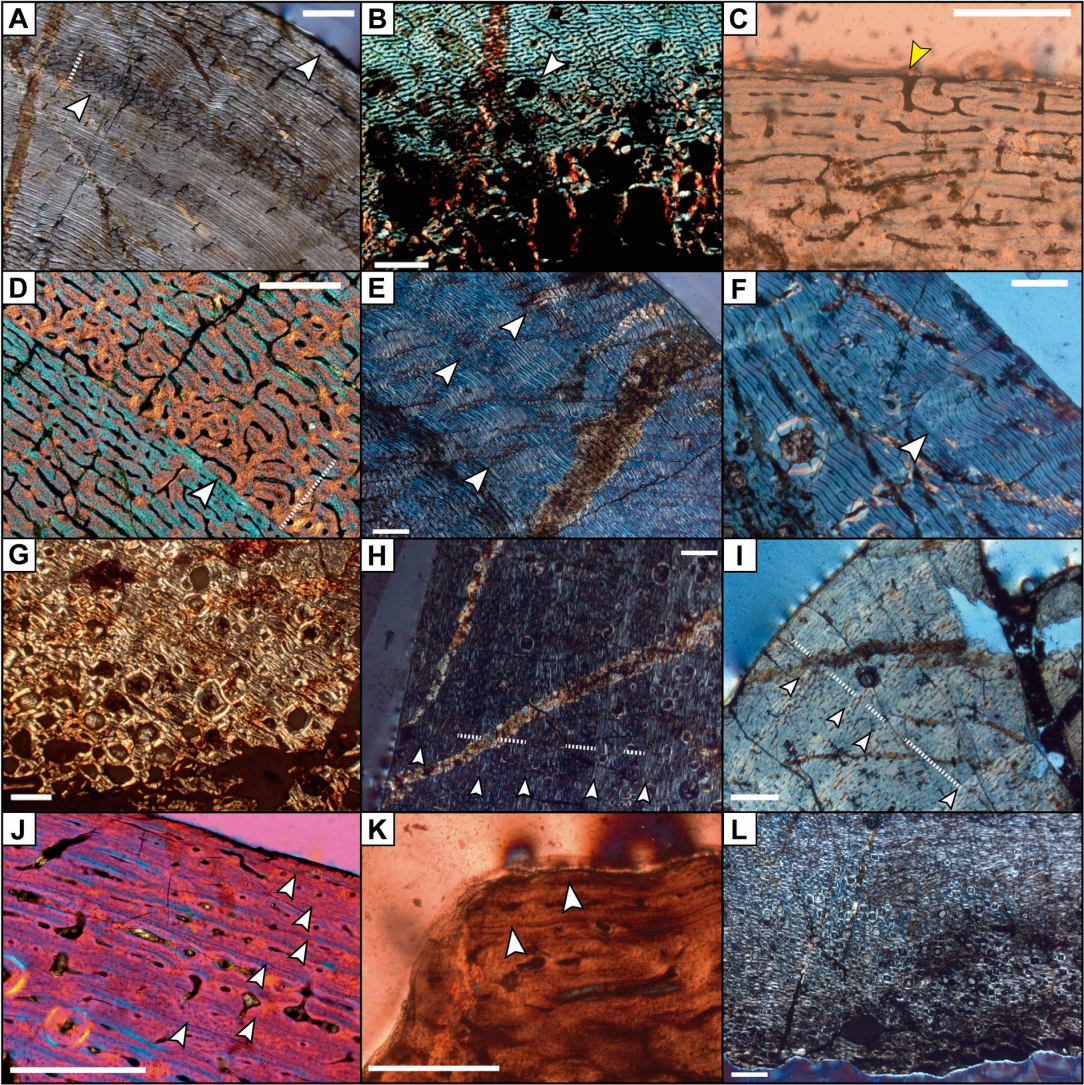
Features of the bone histology in BP femora and tibiae. (A) Bone matrix of the juvenile tibia MCD-4886, showing the last two growth cycles (white arrows) represented by deposition of laminar bone; note the shift from laminar to reticular bone tissue (dotted line) at the beginning of a new growth cycle. (B) Inner cortex of the juvenile tibia MCD-7144; note the incipient remodeling shown by circular erosion cavities (white arrow). (C) Fibrolamellar bone tissue at the periosteal surface of juvenile tibia MCD-4986, with opened vascular canals (yellow arrow) indicating ongoing fast growth. (D) Detail of a LAG in tibia MCD-4986; note the shift from laminar fibrolamellar bone to reticular bone tissue (dotted line). (E) Three growth cycles (white arrows) in the outer region of the cortex of subadult tibia MCD-4728; the large distance between them, the presence of fibrolamellar bone at periosteum, and lack of secondary osteons in the outer cortex, indicate that the animal did not reach maturity and was actively growing at the time of death. (F) Bone matrix of femur MCD-5011 showing a LAG and incipient secondary remodeling near the periosteal surface. (G) Inner cortex of the adult tibia MCD-4920 showing extensive remodeling by secondary osteons (Haversian systems). (H) Growth cycles (dark fast-growing zones, marked with a dotted line, preceding brighter slower-growing zones marked with white arrows) of adult tibia MCD-4920. (I) Closely-spaced growth cycles toward the periosteal surface in adult femur MCD-4723. (J) External fundamental system (EFS) in adult tibia MCD-4918. (K) EFS at periosteum in the adult tibia MCD-5109. (L) Haversian system in the inner cortex of adult tibia MCD-5109. Scale bar equals 1 mm in (A), (B), (E), (F), (G), (H) and (L); 0.5 mm in (C), (D), (J) and (K); and 2 mm in (I).

The tibiae between 390–450 mm long (MCD-4986, MCD-4886, MCD-7144 and MCD-4784) share remarkably similar bone histology. They exhibit juvenile features such as small medullary cavity and bone matrix dominated by plexiform fibrolamellar bone tissue (Fig 11A–11D). Reticular bone is present in the inner areas.

**Fig 12:**
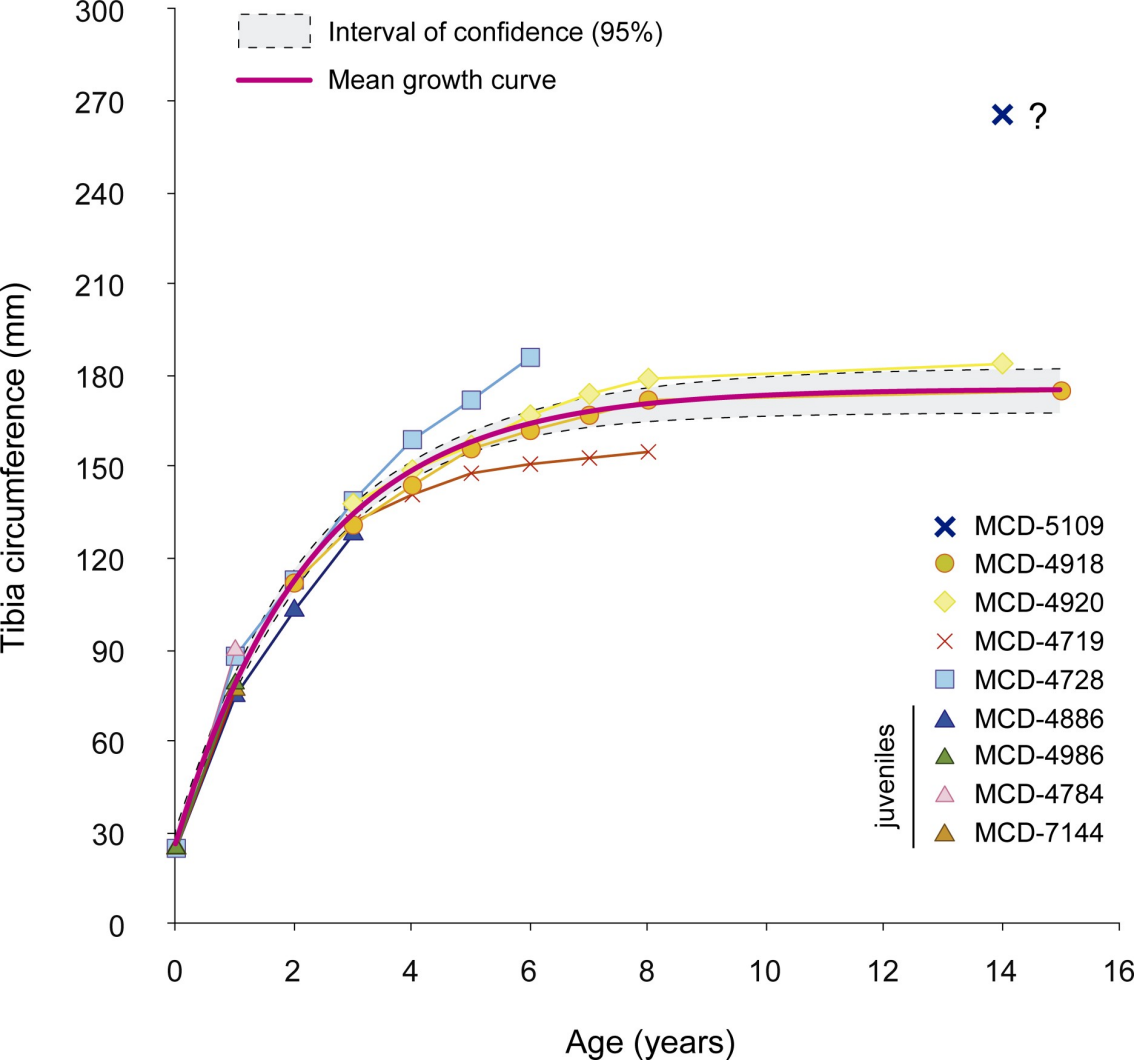
Age-based ontogenetic growth curve of BP. Tibial circumference is used as a proxy for body size (von Bertalanffy's [22] model). Note that the trajectories are similar until age three years. Data are in Table A of S2 Text.

Signs of active reabsorption and early remodeling of bone matrix occur in the form of erosion cavities and secondary osteons. These structures are scattered and located in the innermost region of the bone matrix (Fig 11B). Some specimens reveal open blood vessels in the periosteum that indicate active growth at the time of death (Fig 11C). Near the first rest mark, the tissue is mainly fibrolamellar with longitudinal to circumferential vascularization and laminations that are increasingly fine towards the mark. Rather than a clear rest mark, the abrupt deposition of reticular tissue indicates a sudden change from a slow to a fast growth rate at the beginning of the second growth cycle (Fig 11A and 11D), followed by graded change to fibrolamellar tissue with circumferential, radial and locally longitudinal vascularization. This is very similar to the rest marks shown in Woodward et al. [19]. Specimen MCD-4886 shows two rest marks, with the faint first mark corresponding to that of the younger individuals MCD-4986, MCD-7144 and MCD-4784 (see overlap in the growth curve, Fig 12) and the second mark coinciding in size and shape with the outer cortical perimeter. The robust shape of MCD-4886 can thus be explained by its older age. Increasingly fine lamination towards the periphery (Fig 11A) indicates the forthcoming deposition of a third rest mark in MCD-4886. Overall, the tissue pattern of these specimens corresponds to that described for juvenile ornithopods [18,57] at ages one to three years. Thus, MCD-4986, MCD-4886, MCD-7144 and MCD-4784 represent juvenile individuals. The femora MCD-4708, MCD-4802 and MCD-5104 also share comparable features, so they can be classed as juveniles as well.

The tibiae MCD-4719 and MCD-4728 (550 mm and 720 mm long, respectively) exhibit a more mature bone histology. Both tibiae retain a relatively small medullary cavity; secondary osteon remodeling is more developed than in juveniles, but it is still restricted to the inner-mid region of the cortex. Fibrolamellar bone tissue is present in the outer part of the cortex in MCD-4728, whereas an external fundamental system (EFS) is absent (Fig 11E). The outer margin of MCD-4719 displays a zone with almost exclusively longitudinal vascular channels, a feature related to a decrease in growth rate [58] and suggesting that the development of EFS was imminent. MCD-4728 and MCD-4719 display five and six growth marks, respectively. Together, these features indicate that MCD-4728 belonged to a subadult individual [57], whereas MCD-4719 is likely to have belonged to an early adult. A 720-mm-long femur (MCD-5011) also shares subadult features with MCD-4728 (Fig 11F).

The tibiae MCD-4920 and MCD-4918 (550 mm and 600 mm long, respectively) display more extensive remodeling than other tibiae, with secondary osteons obliterating the inner region of the bone matrix and occasionally reaching the periosteum (Fig 11H–11J). Furthermore, their medullary cavity is expanded. While the above-mentioned juvenile rest marks are followed abruptly by fast-growing reticular tissue that later develops into circumferential and radial vascularization, the transitions are less conspicuous and are graded toward predominantly circumferential and longitudinal vascularization in MCD-4920 and MCD-4918. These transitions frequently are preceded by a line of arrested growth (LAG). In the outermost region of the cortex, these tibiae share a shift from fibrolamellar to a less vascularized bone tissue, with longitudinal blood vessels and closely-spaced LAGs that form an EFS (Fig 11J). This pattern is very similar to the structure described as characteristic for adult individuals of *M*. *peeblesorum* [19,57]. MCD-4920 and MCD-4918 preserve between 12–14 growth cycles/LAGs, including those found in the EFS. These features are characteristic for adult individuals [19,57,59]. The femur MCD-4723 (600 mm long) matches this ontogenetic stage, since it displays a wide medullary cavity and extensive remodeling, although no EFS is observed (Fig 11I). In addition, the large-sized tibia fragment MCD-5109 (≈900 mm estimated total length) also shows an adult pattern. The EFS in the outer region of the cortex of MCD-5109 shows two LAGs within the lamellar bone tissue (Fig 11K). The poor state of preservation limits the identification of growth marks, but at least 12 broader growth cycles can be identified in addition to the two LAGs of the EFS. A dense cloud of Haversian systems occupies most of the bone matrix (Fig 11L). MCD-4920, MCD-4918 and MCD-5109 thus belong to adult individuals.

### Ontogenetic growth trajectories

A growth curve based on the total annual bone circumference of the tibiae provides evidence of initially similar growth trajectories (Fig 12) in juvenile specimens MCD-4986, MCD-7144, MCD-4784, MCD-4886 and in the subadult MCD-4728. There is also a correspondence between growth marks 2 and 3 from MCD-4886 and MCD-4728 and the first visible marks in the older specimens (MCD-4719, MCD-4918 and MCD-4920), indicating that their initial LAGs/growth cycles are lost because of medullary cavity expansion. Superimposition of the transverse sections shows that the growth marks of the various specimens align and are nearly coincident in size, spacing and even shape (insofar as they are not deformed) during ontogeny (Fig C in S2 Text).

The common growth trajectory until age three years suggests that the BP assemblage is a monotaxic population, in which juvenile individuals share the same growth rates. The variance in tibial circumference in the first year is lower in the BP hadrosauroids than in *Maiasaura peeblesorum* (standard deviation is 6.5 in the BP sample for the first year, and 14.5 for *M*. *peeblesorum* according to data from [19]: SI).

Specimens MCD-4719, MCD-4918 and MCD-4920 steadily decelerated in growth from age three through eight years, when asymptotic size and skeletal maturity were attained. The formation of EFS indicates that appositional growth was insignificant from this age onwards (Fig 12). MCD-4728 (subadult) died at the age six years and MCD-4719 (early adult) at age eight years. Thus, the ontogenetic curve confirms the interpretation of MCD-4719 as an early adult tibia, as suggested by its histology. The adult tibiae MCD-4920 and MCD-4918 developed an EFS and died at ages 14 and 15 years, respectively. Tibia MCD-5109 belongs to a much larger adult with an incipient EFS; it appears to be an outlier.

## Discussion

Blanco et al. [15] analysed only a single skeletal element (the dentary) and a small number of BP specimens (six: MCD-4945, MCD-4946, MCD-4963, MCD-5007, MCD-5008 and MCD-5096). They did not compare specimens from a single horizon and locality, but from different horizons (spanning the late early Maastrichtian to the end of the Maastrichtian, an interval of over three million years; [60]) and from different localities in the Iberian Peninsula. Furthermore, their conclusions are supported by morphometrics based on measurements (total dentary length, length and height of the dental battery, inclination of the coronoid process, and apical surface of the coronoid process); counts (total number of alveoli); and morphological features (shape of the coronoid process and its apical portion) that are affected strongly by state of preservation of the specimens. The BP dentaries are affected by the taphonomic biases discussed in the *Taxonomic information and comparison* section. These biases account for differences between our data (e.g., the total dentary length and the sloping of the coronoid process) and those in Blanco et al. [15].

**Fig 13:**
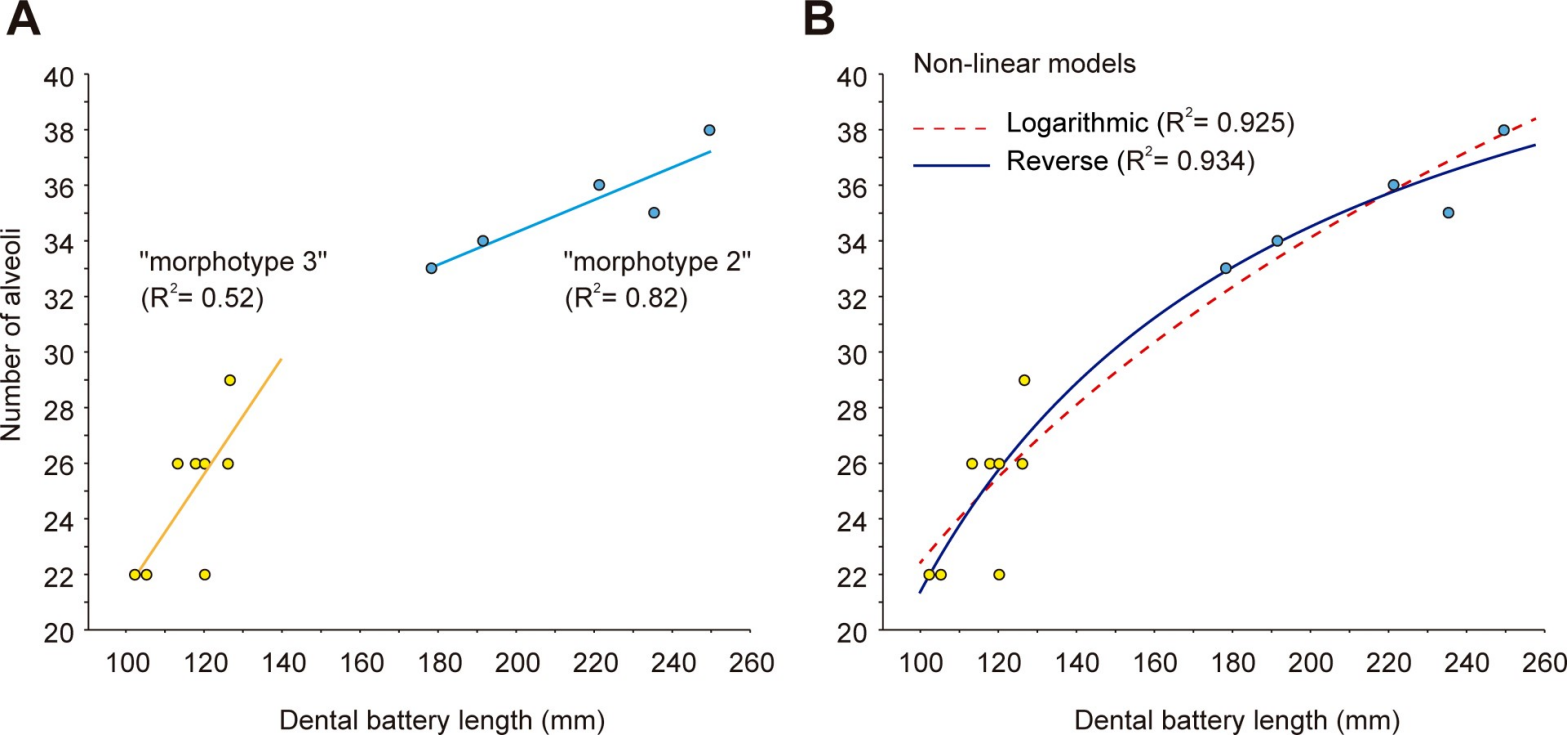
Dental battery length vs. number of alveoli in hadrosauroids from the Iberian Peninsula. (A) Redrawn scatter-plots from Blanco et al. [15]. (B) Non-linear models proposed here for the increase in tooth positions with dental battery size increase. Numerical data were extracted from Blanco et al. ([15]: fig. 6) using ImageJ, and are shown in Table D of S1 Text.

On the assumption that the increase in dental positions (alveoli or tooth families) was linear through ontogeny in hadrosauroids, Blanco et al. [15] calculated linear regressions for the dentary specimens (number of dental positions vs. dental battery length) belonging to dentary morphotypes "2 and 3" (Fig 13A). The differences in the slopes calculated for each dentary morphotype were used to exclude the possibility that the smaller dentaries of “morphotype 3” were juveniles of the larger dentaries ("morphotype 2"). However, the increase in dental positions in living and extinct diapsids is not always linear ([61]: SI, tab. 4).

The non-linear increase in tooth positions with size among hadrosaurids was also recognized by Prieto-Márquez ([62]: 240–241) and Prieto-Márquez et al. ([3]: 21), who noted: "…the number of alveoli varies to some extent even among individuals of comparable size within lambeosaurine species". Even if the increase in dentary tooth positions with increase in dentary size were linear in hadrosauroids, the apparently different trajectories of morphotypes "2" and "3" actually fit a curve better when plotted together (Fig 13B). Two non-linear models were applied to the dentary data from Blanco et al. [15], ascertaining that logarithmic (R^2^ = 0.925) and reverse (R^2^ = 0.934) models fit better than the linear regressions calculated in Blanco et al. [15], which have R^2^ = 0.52 for "morphotype 3" and R^2^ = 0.82 for "morphotype 2". This implies that the rate of accretion of new dental positions within the dental battery of juveniles decreased on attaining adulthood or at least approaching it. Such an interpretation agrees with the asymptotic or sigmoidal growth curve proposed for dinosaurs [18,63–65], and with the ontogenetic curve that we present here. Thus, the number of tooth positions in the dentaries does not support two distinct taxa in the BP sample.

Regression analysis of length to circumference for tibiae and femora (R^2^ = 0.887 for tibiae, R^2^ = 0.933 for femora; Fig 10D and 10E) is consistent with the assumption that the fossil assemblage is monospecific, as occurs with *Maiasaura peeblesorum* (see [19]). Bone histology indicates that the smallest hadrosauroid tibiae from BP correspond to juveniles of a single ontogenetic series (Figs 10 and 12). Histological features of the femora also support a juvenile ontogenetic stage for the smallest bone size-class. Thus, the hypothesized presence of a small-sized adult non-hadrosaurid hadrosauroid taxon in the BP sample proposed by Blanco et al. [15] is not supported by these data. According to our data, the BP dentaries referred to "morphotype 3" by Blanco et al. [15] belong to the small size-class of BP dentaries (Fig 10A) that plausibly are from juvenile individuals. Corroborating this, the nearly vertical coronoid process of the dentary also occurs in some juvenile hadrosaurid specimens (S1 Fig), thus not only in basal hadrosauroids. The BP histograms show that the two BP dentary morphotypes of Blanco et al. [15] probably correspond to the small (juvenile) size-class and the large (adult-subadult) size-class of tibiae and femora (Fig 10A–10C). Taking as a reference the measurements of the dentary and femur of the lambeosaurine *Corythosaurus intermedius* ROM 4670 [53] for scaling, a femur 700 mm long would correspond to a 340-mm-long dentary. Therefore, "morphotype 3" dentaries belong to the same juvenile individuals as the small tibiae and femora.

The high morphological variability among many skeletal elements of the BP sample could be referred to intraspecific variability, especially individual variability, but also to ontogenetic variability and possibly sexual dimorphism. On the basis of a study of the *Iguanodon bernissartensis* material from four stacked bonebeds in Bernissart (Belgium), Verdú et al. [66], combining both morphometric and visual analyses, have shown that features of many postcranial elements (axis, sacrum, caudal vertebrae, scapula, humerus, pollex, ilium, ischium, femur and tibia) that previously were considered to be phylogenetically important among iguanodontian dinosaurs (e.g., [67,68]) actually are intraspecifically variable, and that their variability is individual (age-independent). Individual variability has also been reported among cranial and postcranial elements from subadult and adult individuals of the saurolophine *Brachylophosaurus canadensis* (see [45]).

Some of the variability observed in the BP sample (e.g., the comparatively high robustness of some humeri, tibiae and fibulae) could be related to the age of the individuals to which they belong. Indeed, the tibia MCD-4886 is more robust than others from the same size-class, but it also belongs to an older individual (three vs. two years).

All of the taxonomic information from the whole BP hadrosauroid sample indicates that the bones belong to lambeosaurines. The validity of most of the lambeosaurine species from the French and Spanish Pyrenees (*Pararhabdodon isonensis*, *Arenysaurus ardevoli*, *Blasisaurus canudoi* and *Canardia garonnensis*) is debated [3] because they are based on a few, mostly non-overlapping skeletal elements. For example, the diagnostic features of *P*. *isonensis* and *C*. *garonnensis* are in the maxilla [3], a bone that is only partially preserved in *A*. *ardevoli*. In *A*. *ardevoli*, the only diagnostic feature is in the frontal, which is unknown in *P*. *isonensis*, *B*. *canudoi* and *C*. *garonnensis*; [3]. Furthermore, in *B*. *canudoi* the diagnostic features are in the jugal, which is unknown in *P*. *isonensis* and *C*. *garonnensis* [3] and only partially preserved in *A*. *ardevoli* [38]. The type material of *P*. *isonensis*, *A*. *ardevoli* and *B*. *canudoi* is from the upper Maastrichtian of the Tremp Syncline, Spain. According to Prieto-Márquez et al. [3], *B*. *canudoi* may be a junior synonym of *A*. *ardevoli*, and both may be junior synonyms of *P*. *isonensis*; the designations are retained "pending the finding of sufficiently complete diagnostic overlapping elements" ([3]: 22).

The differences between the BP jugal MCD-5100 and the jugals of *Blasisaurus canudoi* and *Arenysaurus ardevoli*, and those between the BP frontal MCD-4869a and the frontals of *A*. *ardevoli*, support the distinction of the Basturs taxon from these two taxa. A large left maxilla from the Serrat del Rostiar-1 locality of the Tremp Syncline was referred to *Pararhabdodon isonensis* [3]. Serrat del Rostiar-1 horizon is roughly the lateral equivalent to the BP bonebed and is very close to it topographically (Fig 1). The type material of *P*. *isonensis* comes from the Tossal de la Doba hill (the site is reported as Sant Romà d'Abella in the literature), which is only 3.5 km ENE of the BP locality. Although the only maxilla in the BP sample cannot support the attribution to *P*. *isonensis* because of its poor state of preservation, the most parsimonious hypothesis given the present state of knowledge is that the BP bonebed represents an accumulation of *P*. *isonensis* skeletal elements at different growth stages.

However, this hypothesis needs to be confirmed by a larger sample of maxillae, jugals and frontals from the Basturs Poble, Serrat del Rostiar-1, Tossal de la Doba and Blasi localities.

## Conclusions

The BP bonebed is an accumulation of disarticulated lambeosaurine skeletal elements that possibly belong to *Pararhabdodon isonensis*. Juvenile to adult individuals are represented in the sample, with tibia ranging from 390 mm to 940 mm in length. No hatchling remains have been found. Juveniles are over-represented in the sample, and the youngest were two years old. Histologically mature (adult) tibiae are 550–600 mm long and belong to individuals that died when they were 14–15 years old. However, a 550-mm-long tibia belonged to an early adult individual that died at age eight, a six-year-old subadult tibia is 720 mm long and one adult tibia reached an estimated length of 940 mm, revealing individual variation in tibia lengths at skeletal maturity, as in the saurolophine *Maiasaura peeblesorum* (see [19]). Thus, individual maturity cannot be assumed on the basis of bone size alone.

The sample shows a high intraspecific morphological variability in many skeletal elements, including dentaries, dentary teeth, scapulae, humeri, femora, tibiae and fibulae. This suggests the need for caution in choosing characters for phylogenetic analyses, by analogy with the case of *Iguanodon bernissartensis*.

The Basturs Poble bonebed, the Serrat del Rostiar-1 locality and some other hadrosaurid-bearing localities of the Tremp Syncline occur within the C31r magnetochron ([7,31,32]; Fig 1). Consequently, lambeosaurine hadrosaurids were already present and abundant on the Ibero-Armorica Island at the end of the early Maastrichtian. As the type locality of *Pararhabdodon isonensis* is located in the upper Maastrichtian Talarn Formation (within the C29r magnetochron; [7]), this species spans the upper part of the lower Maastrichtian to the upper part of the upper Maastrichtian (upper part of C31r-lower part of C29r).

## Supporting information

S1 Text Measurements of BP dentaries, tibiae and femora. (PDF)

S2 Text Supporting information about bone histology. (PDF)

S1 Fig Straight and nearly vertical coronoid process in juvenile lambeosaurines. (A) Juvenile skull of *Lambeosaurus lambei* (TMP 83.31.02). (B) Dentary of a very young hadrosaurid (TMP 67.20.232). Scale bar equals 10 cm.
(TIF)
