# Supplementary material for: Ontogeny and taxonomy of the hadrosaur (Dinosauria, Ornithopoda) remains from Basturs Poble bonebed (late early Maastrichtian, Tremp Syncline, Spain)
Source: PLoS One. 2018 Oct 31;13(10):e0206287. doi: 10.1371/journal.pone.0206287 (PMC6209292; doi:10.1371/journal.pone.0206287)
Supplement: S1 Text — (PDF) [file pone.0206287.s001.pdf]

S1 Text

Dentaries

With 19 specimens (Text Fig 3), dentaries are relatively well-represented in the sample. Ten specimens are very fragmentary. Four are fragments of left specimens (MCD-4836; MCD-5098; MCD-5097; and MCD-5108a), four are fragments of right specimens (MCD-4726a; MCD-4833; MCD-4942a; MCD-4942b), and one is a fragment with dental sulci (MCD-4944); MCD-4779 might be a fragment of right dentary. Nine dentaries are more complete: three are left (MCD-4963; MCD-5096; and MCD-5012) and six are right elements (MCD-4743; MCD-4744; MCD-4945; MCD-4946; MCD-5007; and MCD-5008). Their estimated total lengths are reported in the S1 Table A.

S1 Table A. BP dentaries estimated total lengths. R and L mean right and left, respectively. Measurements are in millimetres.

Specimen	Length
MCD-5108a (L)	~400-440
MCD-4779 (?R)	~400-440
MCD-4726a (R)	~370-390
MCD-5098 (L)	~370
MCD-4836 (L)	~370
MCD-4944 (?)	~370
MCD-5007 (R)	340
MCD-5097 (L)	~340
MCD-5012 (L)	~220
MCD-5008 (L)	205
MCD-5096 (L)	190
MCD-4743 (R)	190
MCD-4945 (R)	190
MCD-4942a (R)	~190
MCD-4942b (R)	~190
MCD-4833 (R)	~190
MCD-4946 (R)	~185
MCD-4744 (R)	175
MCD-4963 (L)	170

Tibiae and femora

The BP sample includes 23 femora (Text Fig 8). Seven are left femora (MCD-4704, MCD-4723, MCD-4729, MCD-4800, MCD-4802, MCD-5011, MCD-5370) and nine are right femora (MCD-4702, MCD-4708, MCD-4722, MCD-4754, MCD-4801, MCD-4804, MCD-4892, MCD-5107 and MCD-5369). More fragmentary specimens include three right femora (MCD-4742, MCD-4983 and MCD-5104) and four left femora (MCD-4783, MCD-4998, MCD-4941, and MCD-4987). Their total lengths and circumferences are reported in the S1 Table B.

S1 Table B. BP femora total estimated lengths and circumferences. R and L mean right and left, respectively. Measurements are in millimetres.

**Specimen**	**Length**	**Circumference**
MCD-4941 (L)	~800	257
MCD-4742 (R)	~750	-
MCD-5011 (L)	720	239
MCD-5107 (R)	710	243
MCD-4723 (L)	600	212
MCD-4754 (R)	570	210
MCD-5104 (R)	500	162
MCD-4892 (R)	480	149
MCD-4704 (L)	470	145
MCD-4983 (R)	470	-
MCD-4987 (L)	470	142
MCD-4729 (L)	455	160
MCD-4722 (R)	450	144
MCD-4800 (L)	450	148
MCD-4804 (R)	450	139
MCD-4708 (R)	450	135
MCD-4998 (L)	450	-
MCD-4783 (L)	450	-
MCD-4801 (R)	440	136
MCD-4702 (R)	440	122
MCD-4802 (L)	430	131
MCD-5369 (R)	420	135
MCD-5370 (L)	400	133

The BP sample includes 20 tibiae (Text Fig 9A-T). Nine are left (MCD-4705, MCD-4719, MCD-4920, MCD-4799, MCD-4958, MCD-4886, MCD-4986, MCD-5106, MCD-7144) and 11 are right (MCD-4701, MCD-4721, MCD-4728, MCD-4771, MCD-4784, MCD-4882, MCD-4918, MCD-4956, MCD-4796, MCD-5105 and MCD-5109). Right and left specimens with the same total length could belong to the same individual (e.g. MCD-4728 and MCD-4958, right and left, respectively, 730 mm long). Their total lengths and circumferences are reported in the S1 Table C.

S1 Table C. BP tibiae total lengths and circumferences at midshaft. R and L mean right and left, respectively. Measurements are in millimetres. *Measurements estimated based on the tibiae length vs. tibiae circumference regression from the BP tibiae sample (see Text Fig 10E).

Specimen	Length	Circumference
MCD-5109 (R)	940	267*
MCD-4958 (L)	730	191
MCD-4728 (R)	730	201
MCD-4918 (R)	600	175
MCD-4701 (R)	580	160
MCD-4920 (L)	570	184
MCD-4719 (L)	550	157
MCD-5105 (R)	~470	132
MCD-4784 (R)	450	112
MCD-4705 (L)	450	110
MCD-4771 (R)	440	122
MCD-4886 (L)	420	128
MCD-4986 (L)	410	106
MCD-4796 (R)	410	98
MCD-4882 (R)	400	107
MCD-4956 (R)	400	116
MCD-4799 (L)	400	103
MCD-4721 (R)	400	111
MCD-7144 (L)	390	109
MCD-5106 (L)	385	118

S1 Table D. Measurements (mm) of dental battery length and number of alveoli of dentaries found in the Maastrichtian Tremp Formation (southern Pyrenees) used in Fig 13, obtained from Blanco et al. [15].

Specimen	Battery length	Number of alveoli
MCD-4963	102.1	22
MCD-5096	105.1	22
MCD-4946	120.1	22
MCD-4945	113.1	26
MCD-5282	117.7	26
MCD-5181	120.1	26
MCD-5008	126	26
IPS-13092	126.5	29
Blasisaurus MPZ 99/665	178.1	33
MCD-5007	191.3	34
IPS-29920	221.1	36
IPS-82786am	235.1	35
Arenysaurus MPZ2008/258	249.4	38

Reference

15. Blanco A, Prieto-Márquez A, de Esteban S. Diversity of hadrosauroid dinosaurs from the Late Cretaceous Ibero-Armorican Island (European Archipelago) assessed from dentary morphology. Cretaceous Res. 2015; 56:447–457.
