# Supplementary material for: Ontogeny and taxonomy of the hadrosaur (Dinosauria, Ornithopoda) remains from Basturs Poble bonebed (late early Maastrichtian, Tremp Syncline, Spain)
Source: PLoS One. 2018 Oct 31;13(10):e0206287. doi: 10.1371/journal.pone.0206287 (PMC6209292; doi:10.1371/journal.pone.0206287)
Supplement: S2 Text — (PDF) [file pone.0206287.s002.pdf]

S2 Text.

Thin sections - Procedure

The cut surface was exposed using a Buehler Isomet low speed saw, and later polished on a glass sheet coated with carborundum powder, using decreasing particle size (e.g. 600, 800 and 1000 grit). The bone was fixed to a frosted glass slice using ultraviolet curing glue (Loctite 358). The ground section was prepared with a diamond saw (Buehler, PetroThin) to a final thickness of about 100–120 µm. The thin section was polished with a gradient of carborundum (800 and 1200 grit). The slice was dehydrated through a graded series of alcohol baths, cleared in Histo-Clear II during five minutes and finally mounted in DPX mounting medium. In the case of the large tibia MCD-5109 and femur MCD-5011, cores were extracted using an electric power drill mounted in a small-workshop press. After extraction, cores were embedded in epoxy resin before sectioning, using the same methodology explained above.

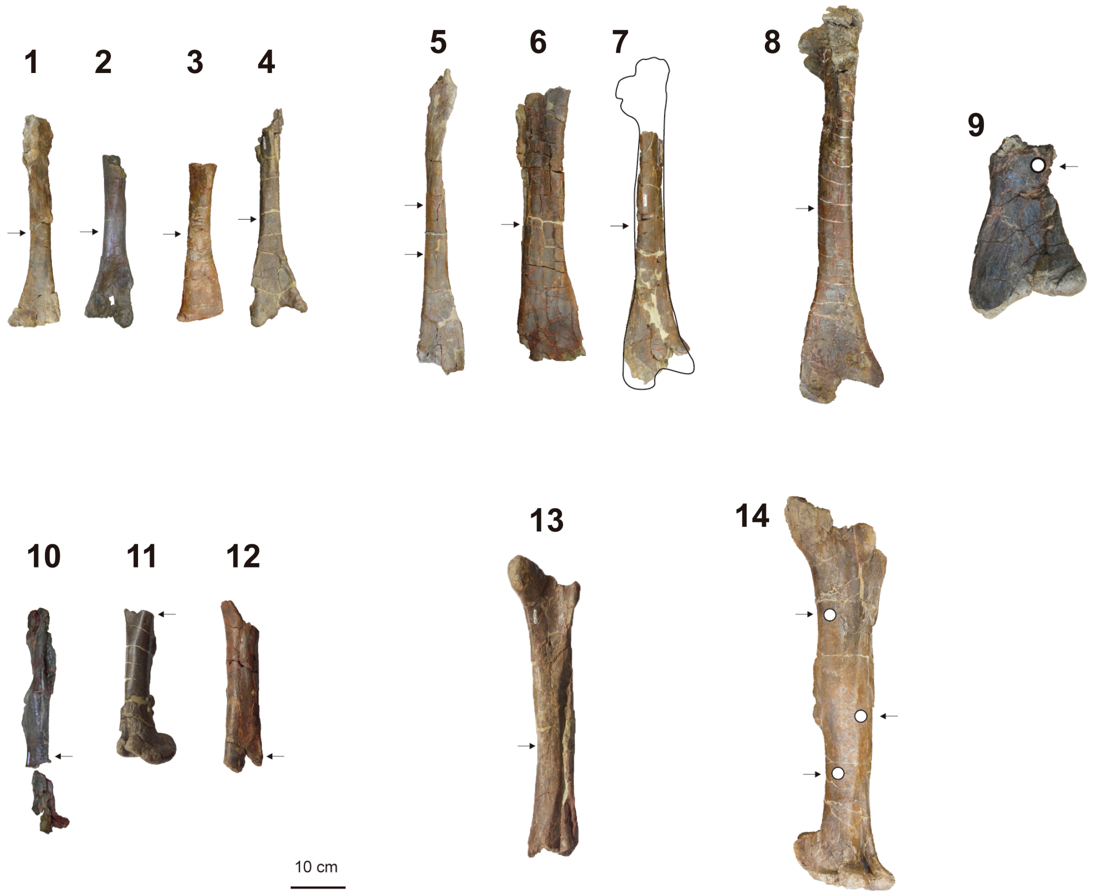

S2 Figure A. Tibiae and femora sampled for histological study. Black arrows indicate the location of each section, while white circles indicate the position of the cores.

Tibiae: (1) MCD-7144 (left). (2) MCD-4986 (left). (3) MCD-4886 (left). (4) MCD-4784 (right). (5) MCD-4719 (left). (6) MCD-4920 (right). (7) MCD-4918 (right). (8) MCD-4728 (right). (9) MCD-5109 (right). Femora: (10) MCD-4802 (left). (11) MCD-4708 (right). (12) MCD-5104 (right). (13) MCD-4723 (left). (14) MCD-5011 (left).

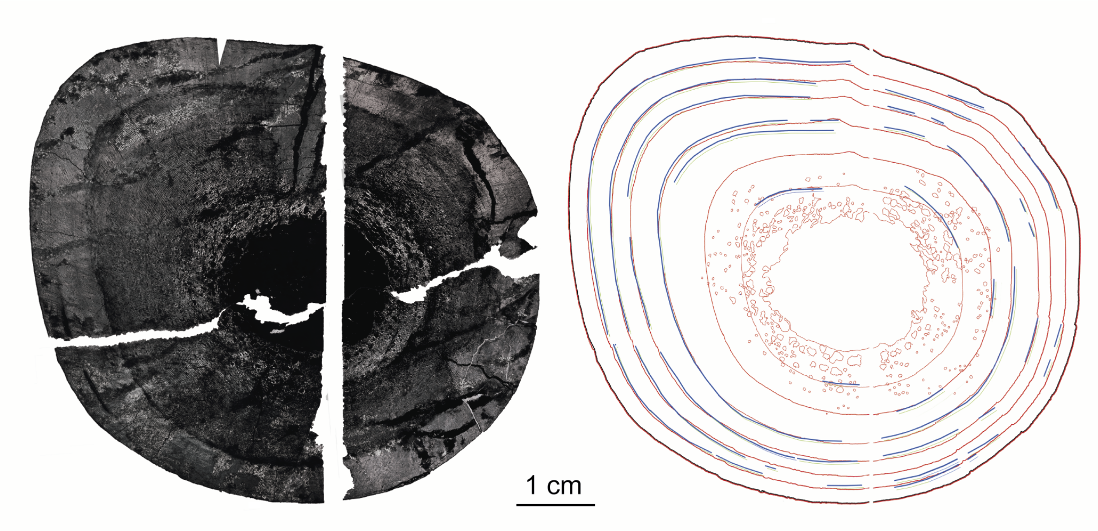

S2 Figure B. Main elements observed in the thin section of tibia MCD-4728. Left, photograph of the section under polarized light; right, schematic view. Blue: LAGs that were identified under polarized light. Red: LAGs that were reconstructed using the external perimeter of the bone.

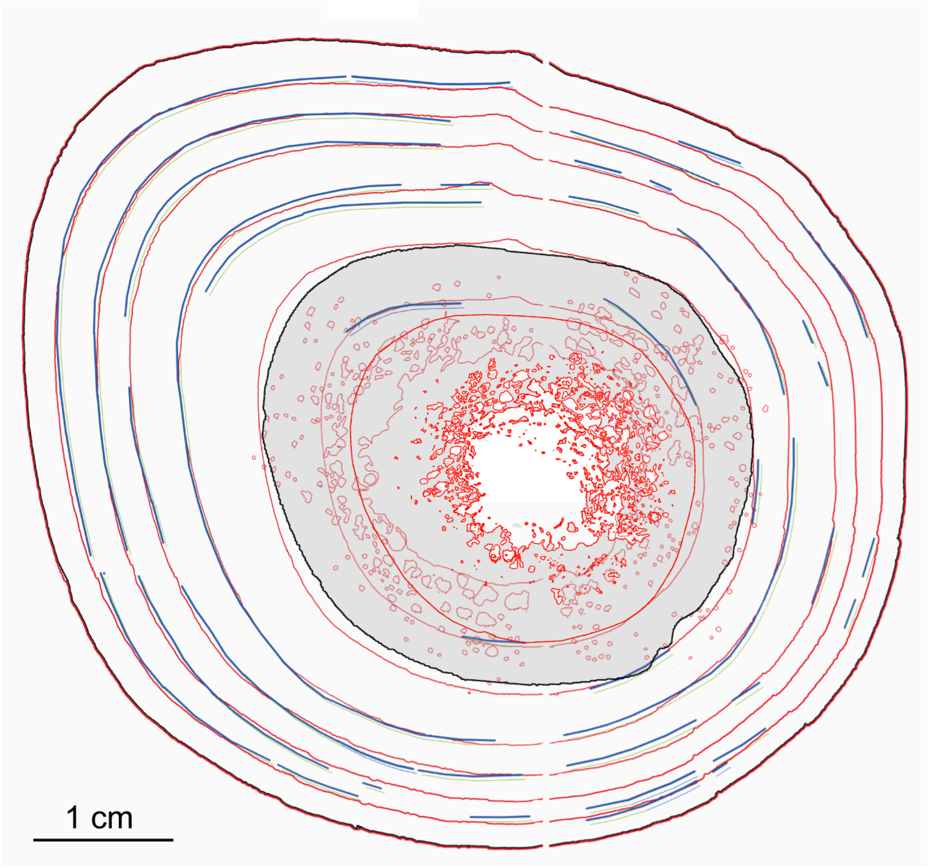

S2 Figure C. Overlapping between tibia sketch MCD-4728 (white) and the flipped (from left to right side) tibia sketch MCD-7144 (grey). Note the similitudes in the reconstructed first growth cycle of MCD-4728 and the first growth cycle of MCD-7144. In addition, it is interesting to see the coincidence between the second growth cycle of MCD-4728 and the periosteal surface of MCD-7144, suggesting that this last individual died just before the development of its second rest mark (see Text).

S2 Table 1. Measurements of the annual LAG circumference in BP tibiae. Reference for perinate tibiae is Cooper et al. [18]. Measurements are in millimetres.

Specimen	Location within the cortex	Circumference
MCD-4986	Perinate tibia (age 0)	25
	LAG 1	79
	Periosteal surface	106
MCD-7144	Perinate tibia (age 0)	25
	LAG 1	77
	Periosteal surface	109
MCD-4784	Perinate tibia (age 0)	25
	LAG 1	90
	Periosteal surface	112
MCD-4886	Perinate tibia (age 0)	25
	LAG 1	75
	LAG 2	103
	LAG 3/Periosteal surface	128
MCD-4728	Perinate tibia (age 0)	25
	LAG 1	88
	LAG 2	113
	LAG 3	139
	LAG 4	159
	LAG 5	172
	LAG 6	186
	Periosteal surface	201
MCD-4719	Perinate tibia (age 0)	25
	LAG 1	Not recorded
	LAG 2	Not recorded
	LAG 3	132
	LAG 4	141
	LAG 5	148
	LAG 6	151
	LAG 7	153
	LAG 8	155
	Periosteal surface	157
MCD-4920	Perinate tibia (age 0)	25
	LAG 1	Not recorded
	LAG 2	Not recorded
	LAG 3	138
	LAG 4	149
	LAG 5	157
	LAG 6	167
	LAG 7	174
	LAG 8 (beginning of EFS)	179

	Periosteal surface (end of EFS)	184
MCD-4918	Perinate tibia (age 0)	25
	LAG 1	Not recorded
	LAG 2	112
	LAG 3	131
	LAG 4	144
	LAG 5	156
	LAG 6	162
	LAG 7	167
	LAG 8 (beginning of EFS)	172
	Periosteal surface (end of EFS)	175
