# Supplementary figures and images for: Ontogeny and taxonomy of the hadrosaur (Dinosauria, Ornithopoda) remains from Basturs Poble bonebed (late early Maastrichtian, Tremp Syncline, Spain)
Source: PLoS One. 2018 Oct 31;13(10):e0206287. doi: 10.1371/journal.pone.0206287 (PMC6209292; doi:10.1371/journal.pone.0206287)

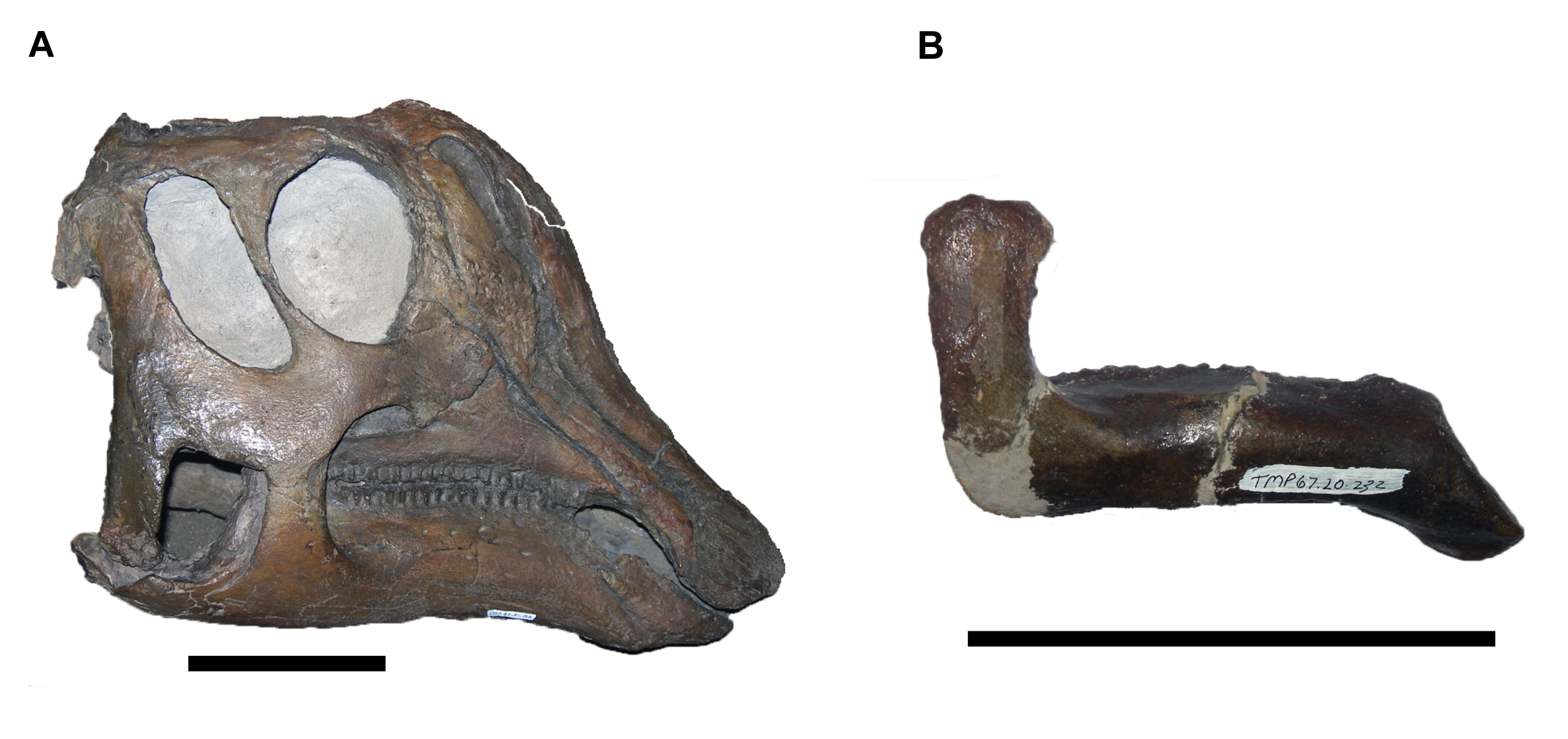

Supplement: S1 Fig — (A) Juvenile skull of Lambeosaurus lambei (TMP 83.31.02). (B) Dentary of a very young hadrosaurid (TMP 67.20.232). Scale bar equals 10 cm. (TIF) [file pone.0206287.s003.tif]
